# Arabidopsis assemble distinct root-associated microbiomes through the synthesis of an array of defense metabolites

**DOI:** 10.1371/journal.pone.0259171

**Published:** 2021-10-26

**Authors:** Enoch Narh Kudjordjie, Rumakanta Sapkota, Mogens Nicolaisen

**Affiliations:** Faculty of Technical Sciences, Department of Agroecology, Aarhus University, Slagelse, Denmark; Fujian Agriculture and Forestry University, CHINA

## Abstract

Plant associated microbiomes are known to confer fitness advantages to the host. Understanding how plant factors including biochemical traits influence host associated microbiome assembly could facilitate the development of microbiome-mediated solutions for sustainable plant production. Here, we examined microbial community structures of a set of well-characterized *Arabidopsis thaliana* mutants disrupted in metabolic pathways for the production of glucosinolates, flavonoids, or a number of defense signalling molecules. *A*. *thaliana* lines were grown in a natural soil and maintained under greenhouse conditions for 4 weeks before collection of roots for bacterial and fungal community profiling. We found distinct relative abundances and diversities of bacterial and fungal communities assembled in the individual *A*. *thaliana* mutants compared to their parental lines. Bacterial and fungal genera were mostly enriched than depleted in secondary metabolite and defense signaling mutants, except for flavonoid mutations on fungi communities. Bacterial genera *Azospirillum* and *Flavobacterium* were significantly enriched in most of the glucosinolate, flavonoid and signalling mutants while the fungal taxa *Sporobolomyces* and *Emericellopsis* were enriched in several glucosinolates and signalling mutants. Whilst the present study revealed marked differences in microbiomes of Arabidopsis mutants and their parental lines, it is suggestive that unknown enzymatic and pleiotropic activities of the mutated genes could contribute to the identified host-associated microbiomes. Notwithstanding, this study revealed interesting gene-microbiota links, and thus represents valuable resource data for selecting candidate *A*. *thaliana* mutants for analyzing the links between host genetics and the associated microbiome.

## Introduction

Plants interact with a vast diversity of microorganisms both above- and belowground, and the outcomes of those interactions may be either beneficial or detrimental to the plant. Essentially, the plant employs a range of strategies such as the action of constitutive and/or induced chemical compounds in combination with the plant innate immune system to assemble its associated microbiota [1]. The plant secondary metabolites glucosinolates (GLS) and flavonoids (FLVs) have been widely studied for several microbiota-mediating and plant protective functions [2]. For instance, GLS from the roots of *Brassica* species were found to inhibit microbial pathogens including *Pseudomonas syringae*, *Alternaria brassicicola*, *Gaeumannomyces graminis*, *Botrytis cinerea*, *Fusarium oxysporum* and *Hyaloperonospora parasitica* [3, 4]. The FLVs are well known for their chemoattractant and signalling function in legume-rhizobia interactions resulting in N-fixation and role in plant-mycorrhizal associations, but also as phytoanticipins [5, 6]. Phytohormones serve as signalling molecules in regulating the innate immune network, and salicylic acid (SA), jasmonic acid (JA), ethylene (ET) and abscisic acid (ABA) act as molecular switches in stimulating inducible defense against biotic and abiotic stresses [7, 8]. Owing to its robust and overarching activation of defense repertoires, the immune system is perceived to affect microbial community structures [1, 9, 10].

The biosynthetic pathways and genes involved in GLS [11, 12], FLV [13–15] and defense signalling [16–18] are well described, and research is directed towards exploiting these pathways to study the links between plant gene functions and microbiome assemblage. Several well-characterized mutants of the model plant, *Arabidopsis thaliana* (hereafter Arabidopsis) have become quintessential for studying those relationships. For example, Badri *et al*. [19] reported effects on microbial communities of a mutation in a plant ATP transporter involved in exudation of plant secondary metabolites, and further concluded that individual plant genes are actively involved in the interaction with microbial communities. By using GLS [20], FLV [19] and benzoxazinoid (BX) mutants [21], the influence of plant defensive secondary metabolites on the plant-associated microbiota has been demonstrated. For example, distinct microbiomes were observed in maize parental lines and their isogenic mutants (*bx1*, *bx2* and *bx6*) carrying disruptions in genes encoding enzymes in different steps of the BX pathway [21]. This study further demonstrated a gatekeeper role of BXs in modulating plant-associated microbiomes associated with plant roots. In other studies, the coumarin-impaired mutants, *myb72-2* and *f6’h1* were used to demonstrate the impact of coumarins on microbial community structures [22, 23]. In addition, studies have used Arabidopsis mutants to examine the influence of phytohormones including fatty acid desaturases (FAD) on microbial community structures [24].

Mechanistic processes at the rhizoplane, including the gating role of plant secondary metabolites and defense signalling molecules (DSMs) could control the assembly of host specific microbiomes. We hypothesized that mutations in pathways for the synthesis of certain secondary metabolites and DSMs disrupt the ability of the plant to assemble an optimal microbiome. Findings from other studies using different experimental systems including either a single or a few mutants have reported contrasting effects of plant metabolites on the plant associated microbial community structures. Moreover, previous studies have relied on in-vitro systems where metabolites were exogenously applied and their effect on microorganisms examined. However, such studies do not always reveal the precise effects of these metabolites in a natural system. Our objective in the present study was to assess root microbiome assembly in natural soils in a range of plant mutants with gene disruption in different steps of defense-related biosynthetic or signalling pathways. For this, we selected a range of well-characterized *Arabidopsis* mutants disrupted in GLS, FLV and DSM synthesis and examined their effects on bacterial and fungal communities in a field soil. The analysis of these mutants using identical soils and growth conditions will provide comparable insights of the effects of these mutations on bacterial as well as fungal community structures, the latter has received little attention in previous studies.

## Materials and methods

### Plant material

**Fig 1:**
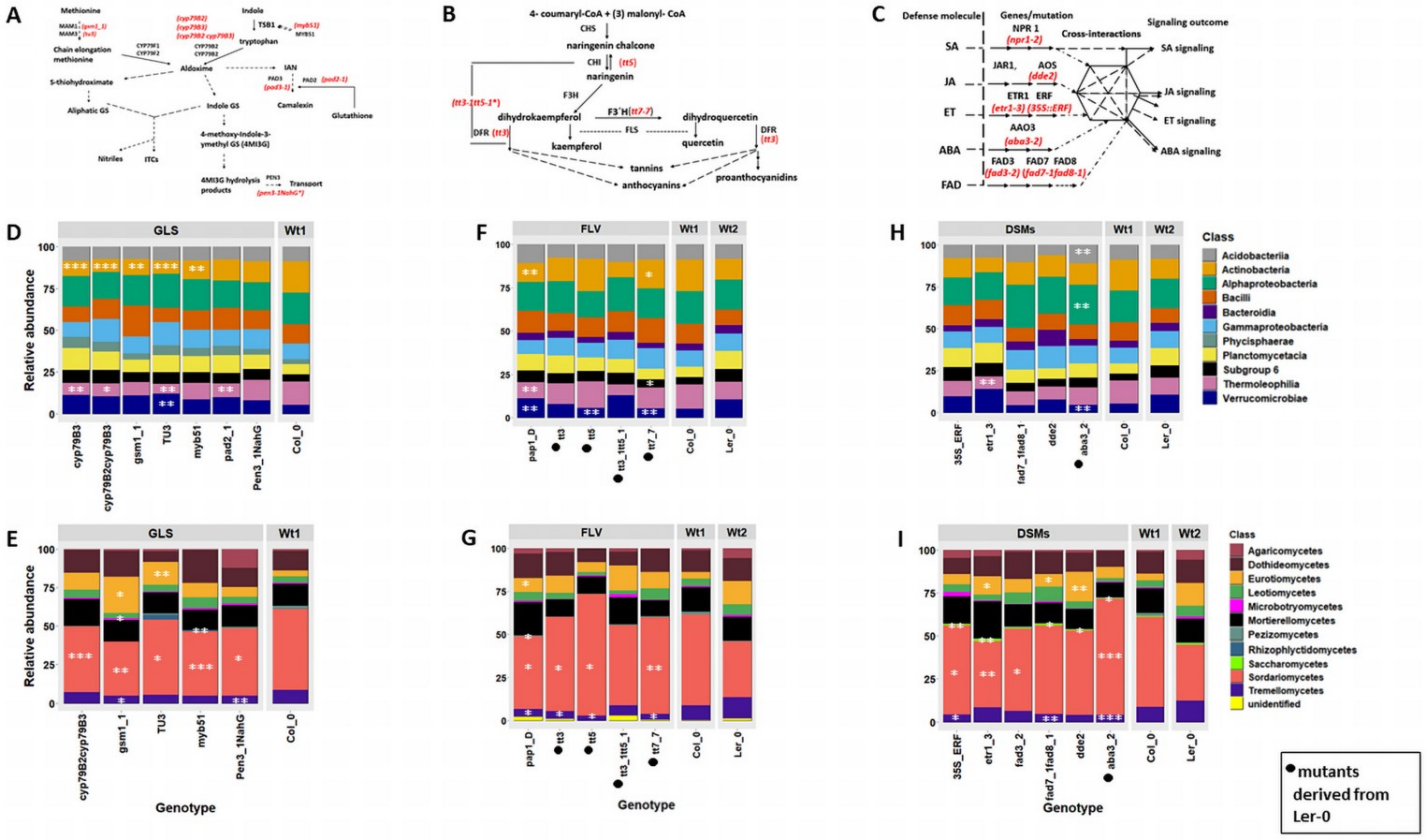
Plant biosynthetic and defense signalling pathways and microbial relative abundances. **A**) Biosynthetic pathway of aliphatic and indolic GLS in Arabidopsis. Adapted from Frerigmann et al, (2014). IAN (Indole-3-acetonitrile), TSB1 (tryptophan synthase beta subunit 1). **B**) The flavonoid biosynthetic pathway. CHI, chalcone isomerase; CHS, chalcone synthase; DFR, dihydroflavonol reductase; F3H, flavonol 3-hydroxylase; FLS, flavonol synthase. *(double mutant). Adapted from Buer et al., (2009, 2010). **C**) The defense signalling pathways. The phytohormones salicylic acid (SA), jasmonic acid (JA), ethylene (ET), abscisic acid (ABA) mediate defense signalling in plants. Fatty acid desaturase (FAD) is also involved in defense including the regulation of JA and SA pathways. Defense signal interactions to fine-tune defense signalling outcomes is also shown. NPR1 (Non-expressor of PR genes1), AAO3 (Abscisic acid synthase), AOS (Allene oxide synthase). Both disrupted genes from which mutants were derived and mutants (indicated in red) are shown. Class-level relative abundances (RAs) of microbial communities observed in Arabidopsis genotypes. **D**) Bacterial and **E**) fungal RAs at class level in GLS mutants and the parental line, Col_0. **F**) Bacterial and **G**) fungal RA at class level in FLV mutants and respective parental lines, Col_0 and Ler-0. **H**) Bacterial and **I**) fungal RAs at class level in DSMs and their respective parental lines, Col_0 and Ler-0. Test of statistical significance was performed by comparing each mutant to their respective background parental lines, and the significantly affected taxa are indicated as *** (P<0.001), ** (P<0.01) and * (P<0.05). ANOVA tests were followed by the Tukey–Kramer *post hoc* test using the Benjamini and Hochberg (BH) FDR for multiple comparisons. Only the most abundant 10 taxa were used in this analysis. Microbial taxa with small effect sizes were removed by filtering (effect size = 0.8). The analysis was performed using the STAMP software (v2.1.3).

We used 21 Arabidopsis mutants and their genetic background lines Col_0 and Ler_0 (Fig 1A–1C; **S1 Table in** S1 File). The parental line Col-0 is a natural accession that is maintained as a homozygous line, while Ler-0 carries X-ray induced mutations in the ERECTA gene [25], resulting in distinct chemical profiles [26], root morphology [27, 28], and resistance against *F*. *oxysporum* [29]. All GLS mutants are in a Col-0 background and similarly for the DSM mutants except for *aba3_2*, which was derived from Ler-0. The flavonoid tt mutants are in a Ler-0 background while pap1_D has Col-0 as its parental line. The GLS (*cyp79B2* and *cyp79B3*), FLVs (*tt3*, *tt5*) and jasmonic acid (*dde2*) mutants were kindly provided by Profs. Judith Bender (Brown University), Wendy Peer (University of Maryland) and Paul Staswich (University of Nebraska), respectively. Other Arabidopsis lines were supplied by the Nottingham Arabidopsis Stock Centre (NASC), UK.

### Experimental design

Arabidopsis seeds were sown in pots (8cm x 8cm x 6cm) with moistened field soil (fine sand 32.2%, coarse sand 52.8%, humus 4.7%, clay 3%, silt 7.3%) [30], pH 5.95, collected from a fallow field at the Jyndevad Research station (N 46° 28’ 20.716’’/E 9° 28’ 45.347’’) in Denmark. Microbiome analysis of the soil revealed high abundance of bacterial phyla Acidobacteria and Proteobacteria and the fungal classes Sordariomycetes and Mortierellomycetes [21]. Each pot represented a biological replicate of individual genotypes and was replicated 5 times for all genotypes. Seeds were stratified and pots completely randomized and maintained in a greenhouse under 2017 summer conditions (**S1 Fig in** S1 File). Seedlings (five plants per pot) were maintained by capillary watering (100 ml) and weed removal every week. Sampling was done after 4 weeks of plant growth, by uprooting each plant and gently shaking roots to remove loosely adhering soils. Roots (with remaining attached fine soils) of the 5 plants in each pot was pooled and placed into 2 ml collection tubes, to represent one replicate. The samples were frozen in liquid nitrogen and stored at -20°C. Subsequently, the samples were lyophilized and ground with 3 sterile metal balls (size 2.88mm) using a Geno/Grinder 2010 at a rate of 1500 rpm for 2 minutes before DNA extraction.

### Sample processing, sequence analysis and statistics

Sample DNA extraction and library preparation were essentially performed as previously described [21]. Briefly, we extracted DNA using the PowerLyzer^™^ Power Soil^®^ DNA Isolation Kit (Mo Bio Laboratories, Carlsbad, CA, USA). The bacterial primers S-D-Bact-0341-b-S-17, 5′-CCTACGGGNGGCWGCAG-3′ and S-D-Bact-0785-a-A-21, 5′-GACTACHVGGGTATCTAATCC-3′ [31] and the fungal primers fITS7, 5′-GTGARTCATCGAATCTTTG-3′ and ITS4, 5′-TCCTCCGCTTATTGATATGC-3′ [32] were used to amplify the V3/V4 region of the bacterial 16S rRNA and the fungal internal transcribed spacer 2 (ITS2) region, respectively. A dual indexing strategy was used, and PCR conditions were as described [21]. For detailed library and PCR conditions see **Additional file 1 in** S1 File. Samples were sequenced using the Illumina MiSeq platform at Eurofins MWG (Ebersberg, Germany). All sequence files were deposited in the NCBI Sequence Read Archive (SRA) under the accession number PRJNA579829.

Sequence analysis including demultiplexing, operational taxonomic units (OTUs) clustering at 97% similarity cutoff value, chimera detection and removal, and OTU table creation were performed using VSEARCH version 2.6 [33], as described in [21]. Taxonomy assignments were carried out in QIIME version 1.9 [34], respectively using SILVA 132 [35] and UNITE version 7.2 [36] reference databases for bacterial and fungal OTUs. Downstream data exploration and visualization was performed in the R statistical package [37] using the ggplot2 (v3.3.2) package. Microbial community diversity estimations including alpha and beta diversities, species richness and dissimilarity were performed using the ‘vegan’ package [38] and ‘phyloseq’ [39]. We applied a minimum cutoff of 700 and 500 reads respectively, for bacteria and fungi and the reads and OTU distribution were visualized (**S2 Fig in** S1 File). Following this, samples with less than 3 replicates were removed and OTUs represented in less than 3 samples in the total dataset were also excluded prior to downstream analysis.

To determine statistically significant differences in taxonomic profiles, we performed a multiple group analysis by comparing sequences assigned to different class level taxa (top 10) in mutants and their respective parental lines (Col_0 or Ler-0) using the STAMP software v2.1.3 [40, 41]. Genotypes were compared by ANOVA, followed by the Tukey–Kramer *post hoc* test (p < 0.05) using the Benjamini and Hochberg (BH) FDR for multiple comparisons. Class taxa with small effect sizes were removed by filtering (effect size = 0.8).

Bacterial and fungal alpha diversities (observed and Shannon) were estimated by rarifying the OTU table 100 times at a depth of 700 reads for bacteria and 500 reads for fungi and the mean of the diversity estimates of 100 trials was used. To identify the effect of host genotype on alpha diversity, we partitioned the data for each mutant and parental line and performed Kruskal-Wallis test, followed by pairwise Wilcoxon rank sum tests with Benjamini-Hochberg correction. The OTU tables were transformed to relative abundances (RAs) for beta diversity analysis for each genotype groups (GLS, FLV and DSM mutants), and further visualized using unconstrained principal coordinates analysis (PCoA). Permutational multivariate analysis of variance (PERMANOVA) based on a Bray-Curtis dissimilarity matrix was carried out by using 1000 permutations to detect significant differences in the overall bacterial and fungal community composition, using the “adonis” test from the “vegan” package [38]. We further confirmed significant genotypic variations on microbial community structures using generalized linear models (GLMs), where genotype was fitted to each OTU using the mvabund package (function *manyglm* with a negative binomial distribution) [42]. The likelihood-ratio test was used, and p-values were adjusted for multiple testing. To further analyse specific genotype effects, we split the data for each mutant and parental line and performed PERMANOVA analysis. We performed differential analysis using DESeq2 (version 1.22.2) [43] to determine bacterial and fungal taxa that were significantly different in mutants and parental lines.

## Results

We studied microbiome composition in Arabidopsis plants carrying mutations in specific steps of the glucosinolates (GLS), flavonoids (FLV) and defense signaling molecules (DSM) pathways (Fig 1A–1C). The bacterial community profiling yielded a total of 717,379 sequence reads clustering into 6,471 OTUs while the fungal profiling yielded 703,675 sequences which clustered into 344 OTUs after quality filtering. The sequence read statistics are provided (**S2 Table in** S1 File**)**.

### Microbial abundance is affected in most mutants

Bacterial and fungal class relative abundances were distinct in the GLS, FLV and DSM mutants compared to parental lines (Fig 1). We found the most highly significant differences (P<0.001) in the mean abundance of reads belonging to the bacterial classes Actinobacteria, Thermoleophilia and Verrucomicrobiae in the GLS upstream mutants *cyp79B3*, *cyp79B2cyp79B3* and *tu3* (Fig 1D). Both *cyp79B3 and cyp79B2cyp79B3* have upstream mutations that lacks the ability to form aldoxime from tryptophan and, thus, are deficient in indolic GLS [44, 45], while *tu3* lacks C_6_, C_7_, and C_8_ aliphatic glucosinolates [12]. Similarly, the fungal class Eurotiomycetes was highly enriched in *gsm1_1* and had the least abundance in Col_0 (Fig 1E). Dothidiomycetes was strongly enriched in *myb51*(impaired in IGS and camalexin synthesis) (Fig 1E).

A multiple test analysis revealed significant enrichment of a number of bacterial taxa in FLV mutants *pap1_D (FLV* overexpressed mutant) and *tt7_7* compared to their parental lines. Verrucomicrobiae was strongly enriched in *tt5* and *tt7_7* (deficient in flavonoid 3’-hydroxylase activity and lacking orthodihydroxy flavonoids such as quercetin and kaempferol) compared to Ler_0 (Fig 1F). Similarly, we found significant differences in the relative abundances of Sordariomycetes and Tremellomycetes among the mutants (Fig 1G). In the DSM mutant *aba3_2*, Acidobacteria and Alphaproteobacteria were enriched while Verrucomicrobiae was depleted compared to the parental line Ler_0 (Fig 1H). Thermoleophilia was strongly depleted in *etr1_3*. Sordariomycetes was enriched, while Tremellomycetes was depleted in *aba3_2* (Fig 1I). The relative abundance of other fungal taxa including Eurotiomycetes and Leotiomycetes were also significantly affected.

### GLSs, FLVs and DSMs all affect root microbial diversity

**Fig 2:**
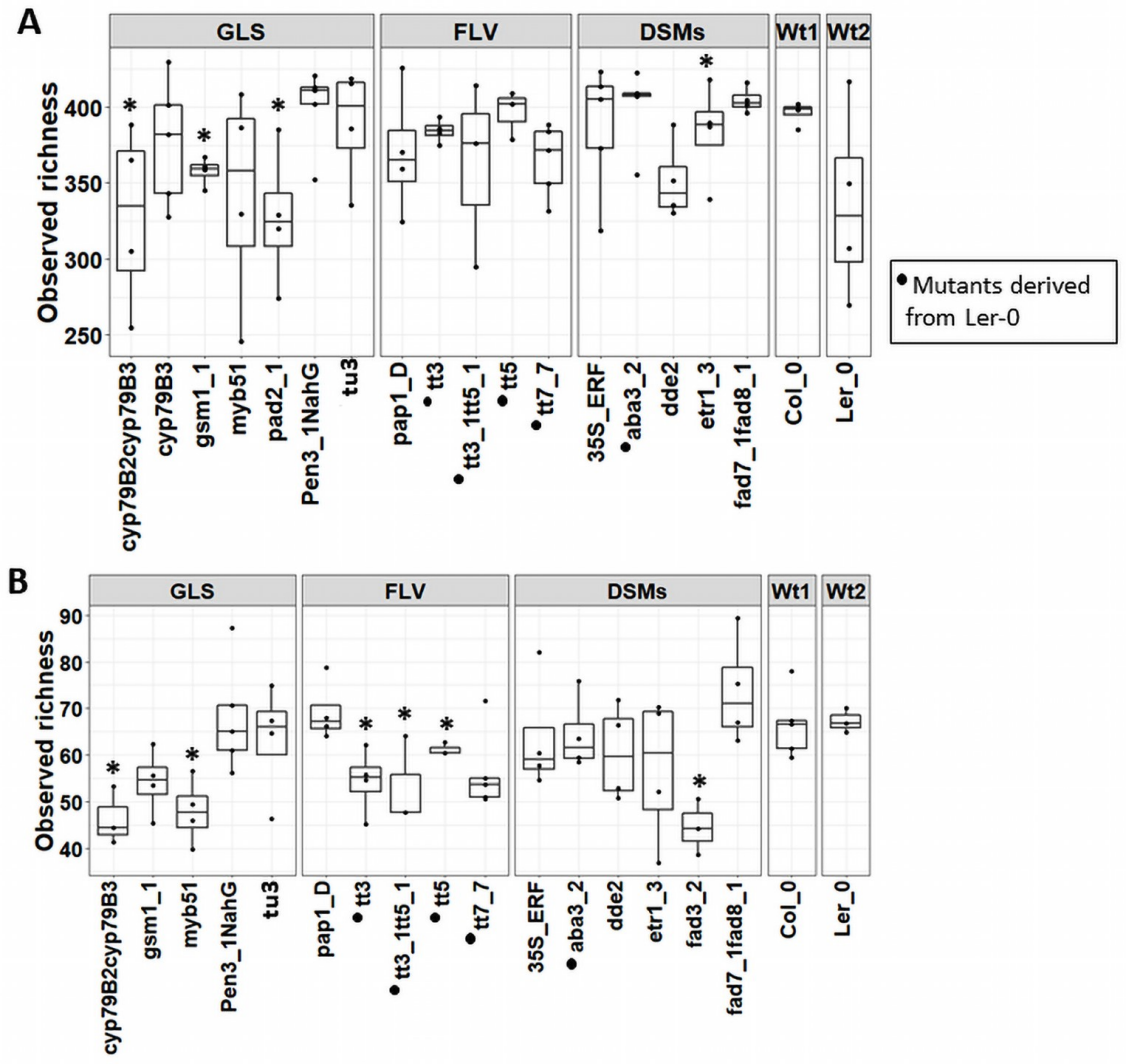
Alpha diversity (observed) of bacterial (A) and fungal (B) communities in roots of Arabidopsis secondary metabolite and signalling mutants and their parental lines (Col_0 and Ler-0). Significant differences in alpha diversity in secondary metabolite and signalling mutants and their parental lines are indicated as *, **, and *** for P <0.05, P<0.01 and P<0.001 respectively. The analysis was performed using the Kruskal-Wallis test.

We found significant differences in bacterial observed richness (P<0.05) in the GLS mutants *cyp79B2cyp79B3*, *gsm1_1* and *pad2_1* and in the DSM mutant *dde2* (Fig 2, **S3 Table in** S1 File). Shannon diversity was also significantly different (P<0.05) among the *pad2_1* and *dde2* mutants (**S3A Fig in** S1 File, **S3 Table in** S1 File). Bacterial alpha diversity was not significantly different in any of the FLV lines; however, the observed richness was higher in these mutants (the tt lines) compared to Ler_0. For fungi, the observed richness was significantly lower (P< 0.05) in the GLS *cyp79B2cyp79B3*, *myb51*, the FLVs *tt3*, *tt3_1tt5_1*, *tt5* and the DSM mutant *fad3_2* compared to Ler_0 (Fig 2, **S3 Table in** S1 File). In addition, Shannon diversity was significantly different (P<0.05) in the DSM mutant *aba3_2* (**S3B Fig in** S1 File, **S3 Table in** S1 File).

**Fig 3:**
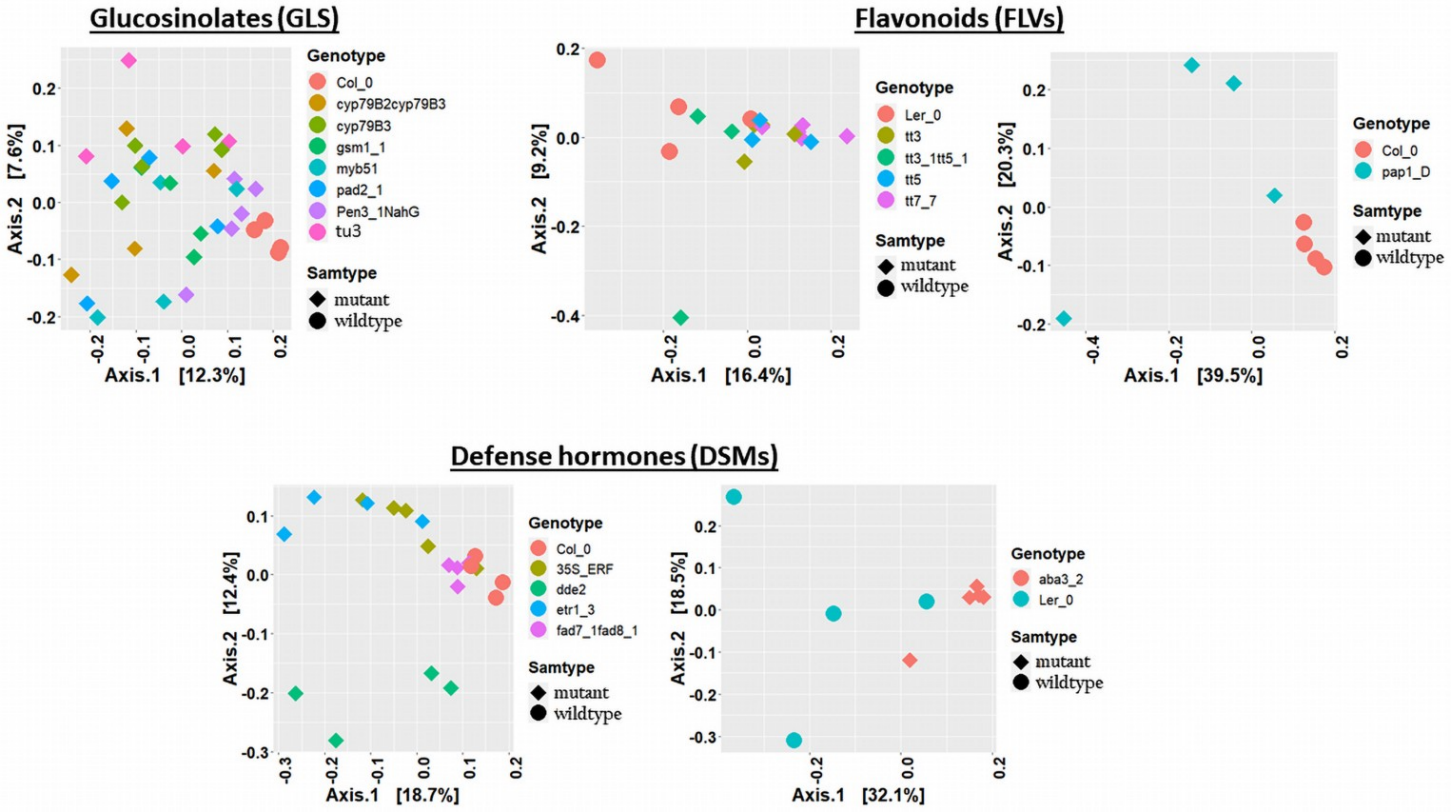
Principal coordinate analysis (PCoA) plots of bacterial communities in Arabidopsis secondary metabolite and signalling mutants, and their parental lines. PCoA of bacterial communities in GLS (**A**), FLV (**B**) and DSM (**C**) genotypes. Individual genotypes and sample groups are shown in different colours and shapes.

**Fig 4:**
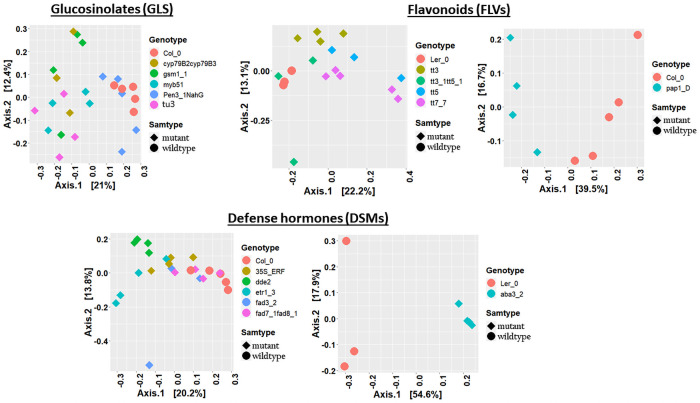
Principal coordinate analysis (PCoA) plots of fungal communities in Arabidopsis secondary metabolite and signalling mutants, and their parental lines. PCoA of fungal communities in GLS (**A**), FLV (**B**) and DSM (**C**) genotypes. Individual genotypes and sample groups are shown in different colours and shapes.

Bacterial beta diversity analysis and visualization using PCoA ordination plots showed a clear separation of GLS mutants from Col_0 (Fig 3A). PERMANOVA revealed significant differences on the bacterial communities (Adonis, bacteria: R^2^ = 0.31, P< 0.001; Table 1; **S4 Table in** S1 File,). We likewise observed clustering in the fungal PCoA plots, except that Col_0 and *pen3_1_NahG* (impaired in both IGS and SA) clustered together (Fig 4A), and the GLS mutation also significantly affected fungal communities (Adonis, R^2^ = 0.37, P< 0.001, Table 1; **S5 Table in** S1 File). The *tu3* mutant explained the highest proportion of the variation on both bacterial (Adonis, R^2^ = 0.34, P<0.01, Table 1) and fungal (Adonis, R^2^ = 0.41, P<0.01, Table 1) communities. The *pen3_1_NahG* mutant, which is disrupted in both metabolism and transport of tryptophan-derived secondary metabolites and SA synthesis, expectedly had a strong effect on bacterial communities. However, *pen3_1_NahG* line also carries mutation in PEN3 gene known for cell-wall defense enhancement [46], thus, its effect on the microbiome should be interpreted cautiously. Similarly, PCoA ordination plots showed clustering in the FLV mutants and parental lines in both bacterial and fungal datasets (Figs 3B and 4B) and significant differences were confirmed by PERMANOVA for bacterial (Adonis, R^2^ = 0.36, P< 0.001, Table 1; **S4 Table in** S1 File)) and fungal (Adonis, R^2^ = 0.38, P<0.01, Table 1; **S5 Table in** S1 File) community structures. Further PERMANOVA analysis using datasets consisting of individual mutants and their parental lines revealed the highest effects of *tt3_1_tt5_1* and *tt7_7* mutants on bacterial (Adonis; R^2^ = 0.28, P<0.05, Table 1) and fungal (Adonis; R^2^ = 0.33, P<0.05, Table 1) communities, respectively. A minor but significant effect of *tt3* (Adonis; R^2^ = 0.31, P<0.05, Table 1) and *pap1_D* (Adonis; R^2^ = 0.33, P<0.05, Table 1) was detected on the fungal communities.

**Table 1:** Permutation analysis of variance (PERMANOVA) for individual mutants and background parental lines. Adonis tests were based on Bray-Curtis dissimilarity matrices for both bacterial fungal communities using 1000 permutations.

Dataset/ Factor	Genotype description	Bacteria (R^2^)	Fungi (R^2^)
GLS (Mut1)	GLS mutants and parental lines	0.31***	0.37***
FLV (Mut2) data	FLV mutants and parental lines	0.36***	0.38**
DSM (Mut3)	Defense signalling mutants and parental lines	0.39***	0.40***
**GLS**			
Col_0_*cyp79B3*	IGS partial disruption)	0.26**	--
Col_0_*cyp79B2cyp79B3*	Lacks IGS and camalexin (Blocked in the production of I3AOx)	0.28*	0.33*
Col_0_*myb51*	IGS synthesis disruption	0.26*	0.30*
Col_0_*gsm1_1*	Reduced amounts of many aliphatic glucosinolates	0.26*	0.34*
Col_0_*tu3*	Deficient in aliphatic GLS with heptyl and octyl core groups	0.34*	0.41*
Col_0_ *pad2-1*	Partially blocks camalexin	0.28*	--
Col_0_ *pen3_1_NahG*	Disruption in both IGS synthesis and SA signalling pathways.	0.20**	0.16*
**FLV**			
Ler-0_*tt7_7*	Deficient in flavonoid 3’-hydroxylase activity and lacks orthodihydroxy flavonoids such as quercetin and kaempferol	0.23*	0.33*
Ler-0_*tt3*	Excess quercetin, kaempferol	--	0.31*
Ler-0_*tt5*	Low-level quercetin production	n.s	n.s
Ler-0_ *tt3_1tt5_1*	Double mutation, disruption of the synthesis of brown pigment	0.28*	n.s
Col_0_*pap1_D*	Overexpressed (anthocyanin) mutant	n.s	0.35*
**DSMs**			
Col_0_*dde2*	JA deficient	0.33*	0. 41*
Ler-0_ *aba3_2*	ABA deficient	0.24*	0.54*
Col_0_ *etr1_3*	Ethylene responsive	0.33*	0.32*
Col_0_*35S*::*ERF*	Ethylene (overexpressed)	0.19*	0.24*
Col_0_ *fad3_2*	Fatty acid desaturase (FAD) deficient	--	0.25*
Col_0_ *fad7_1fad8_1*	FAD double mutation	0.31*	--

Significance of test indicated as

*** for p<0.001,

** p <0.01,

*p<0.05 and R^2^ for the proportion of variation explained. I3AOx (Indole-3-aldoxime) IGS (indole glucosinolate).

^--^ not computed due to few sample (<3) replicates and n.s (not significant).

The DSM mutants and their parental lines likewise showed clear clustering of bacterial and fungal communities (Figs 3C and 4C) and were further confirmed to be significantly different (bacteria: Adonis; R^2^ = 0.39, P<0.001, Table 1; **S4 Table in** S1 File, and fungi: Adonis; R^2^ = 0.40, P<0.001, Table 1; **S5 Table in** S1 File). PERMANOVA performed on the individual mutants and their parental lines showed that the highest proportion of variation was contributed by the *dde2* and *etr1*_*3* mutations on the bacterial communities (*dde2*: Adonis; R^2^ = 0.33, P<0.001; *etr1_3* Adonis; R^2^ = 0.33, P<0.001, Table 1), and the *aba3_2* mutation on the fungal communities (Adonis; R^2^ = 0.54, P<0.001, Table 1).

### GLS, FLV and DSM mutations specifically enrich or deplete microbial taxa

**Fig 5:**
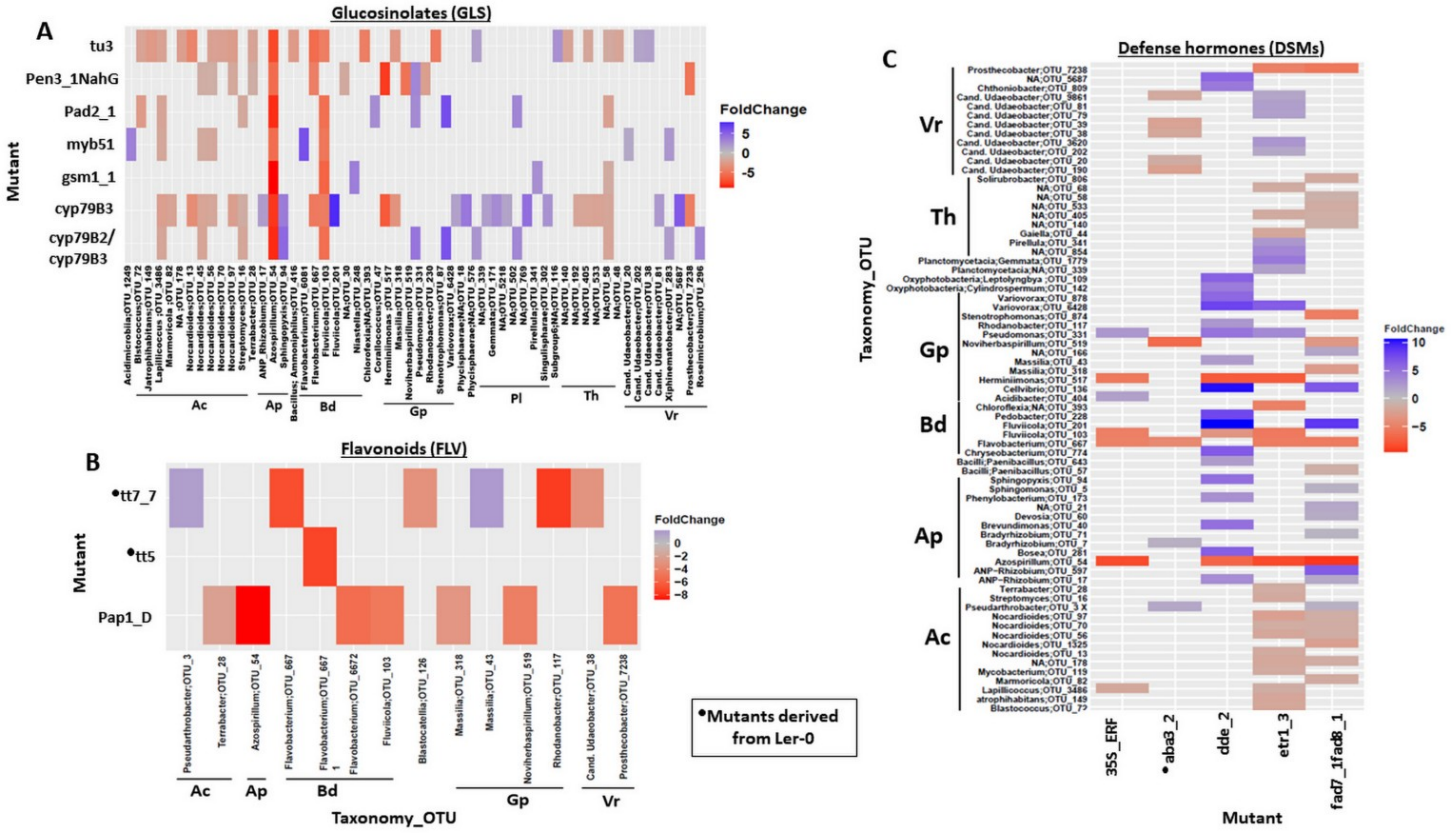
Heat map of differentially abundant bacterial OTUs identified by comparing secondary metabolite and signalling mutants and their parental lines. **A)** Heat map of differentially abundant bacterial OTUs identified by comparing GLS mutants and parental lines. **B**) Heat map of differentially abundant bacterial OTUs identified by comparing FLVs mutants and parental lines. **C**) Heat map of differentially abundant bacterial OTUs identified by comparing DSMs mutants and parental lines. Foldchanges are indicated in blue and red respectively for parental lines and mutants. *ANP-Rhizobium* (*Allorhizobium-Neorhizobium-Pararhizobium-Rhizobium*), *Candidatus Udaeobacter* (*Cand*. *Udaeobacter*), *Candidatus Xiphinematobacter* (*Cand*. *Xiphinematobacter*), Ac (Actinobacteria), Ap (Alphaproteobacteria), Bd (Bacteroidetes), Gp (Gammaproteobacteria), Pl (Planctomycetes), Th (Thermoleophilia), Vr (Verrucomicrobiae). Analysis was performed using DESeq2 package.

Next, we performed differential analysis to determine bacterial and fungal OTUs (bOTUs and fOTUs, respectively) that had significantly different relative abundances between mutants and parental lines. Most of the differentially affected genera belong to dominant microbial taxa and are mostly enriched than depleted when secondary metabolite and signaling pathways are altered, except for flavonoid mutations on fungi communities (**S6 Table in** S1 File). For GLS, the highest numbers of differentially abundant bOTUs were observed in upstream mutated lines *tu3* and *cyp79B3* (Fig 5A; **S7 Table in** S1 File). Bacterial phyla Actinobacteria, Alphaproteobacteria and Bacteroidetes were among the most highly enriched taxa in many of the GLS mutants. bOTUs assigned to the genera *Nocardioides* and *Azospirillum* were the most highly enriched in several GLS mutants. Other significantly enriched genera included *Streptomyces* and *Fluviicola* in mutants such as *tu3*, *myb51* and *cyp79B3* (Fig 5A; **S7 Table in** S1 File). Planctomycetes genera were mostly depleted in *cyp79B3*. *Massilia* and *Flavobacterium* were also significantly enriched in *cyp79B3* and *tu3*. These results indicate that both indolic and aliphatic GLSs regulate microbial members.

We found the lowest number of significantly affected bOTUs in the FLV mutants, with most of these taxa enriched in *tt7_7* and *pap1-D* (Fig 5B; **S8 Table in** S1 File). bOTUs belonging to *Flavobacterium* were enriched in the FLV mutants, and *Rhodanobacter* and *Azospirillum* were significantly enriched in *tt7_7* and *pap1-D*, respectively. The DSM mutant *dde2* was mostly depleted in Proteobacteria and Bacteroidetes genera while Actinobacteria genera were enriched in *etr1_3* and *fad7_1fad8_1*. Other mutants such as *35S*::*ERF*, *aba3_2* and *dde_2* were enriched in *Azospirillum*, *Flavobacterium* and *Fluviicola* (Fig 5C; **S9 Table in** S1 File).

**Fig 6:**
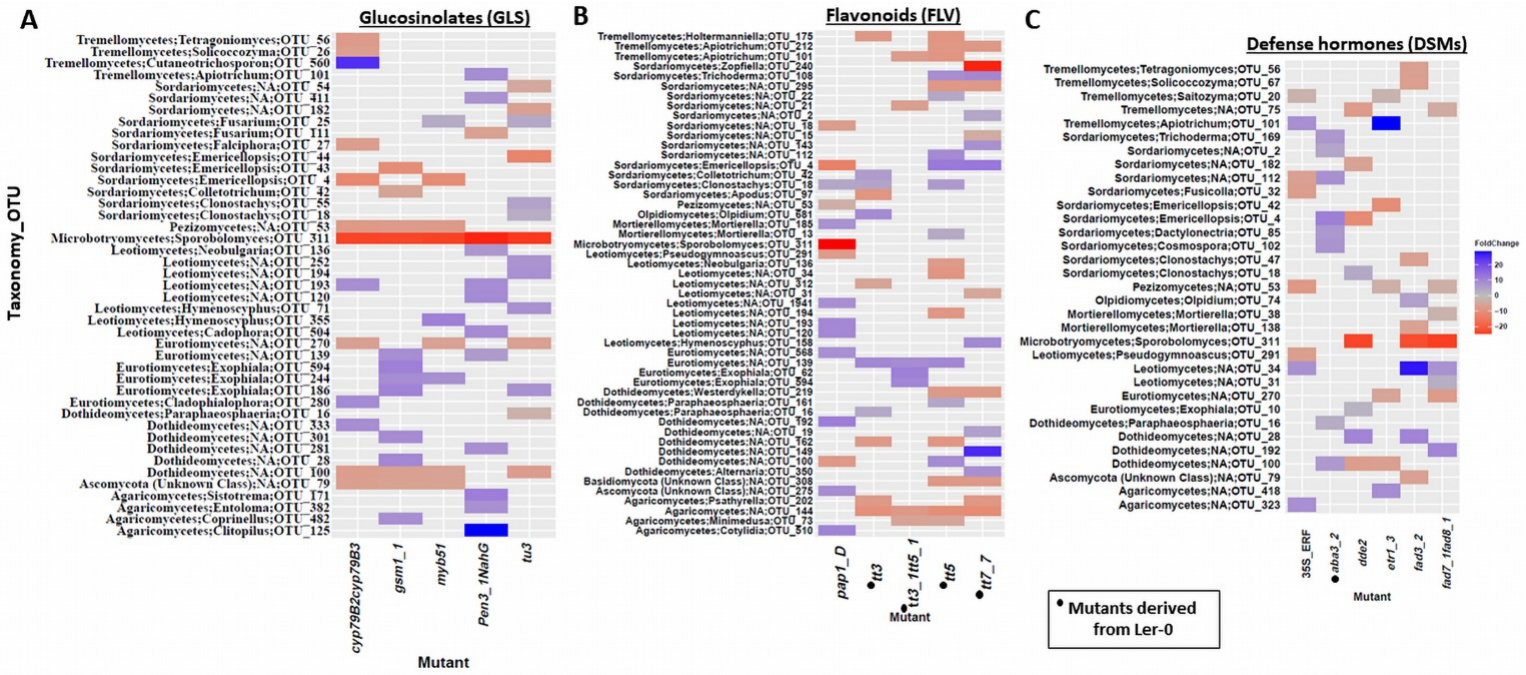
Heat map of differentially abundant fungal OTUs identified by comparing secondary metabolite and signalling mutants and their parental lines. **A)** Heat map of differentially abundant fungal OTUs identified by comparing GLS mutants and parental line. **B**) Heat map of differentially abundant fungal OTUs identified by comparing FLVs mutants and parental line. **C**) Heat map of differentially abundant fungal OTUs identified by comparing DSMs mutants and parental line. Foldchanges are indicated in blue and red respectively in parental lines and mutants. *ANP-Rhizobium* (*Allorhizobium-Neorhizobium-Pararhizobium-Rhizobium*), *Candidatus Udaeobacter* (*Cand*. *Udaeobacter*), *Candidatus Xiphinematobacter* (*Cand*. *Xiphinematobacter*). The analysis was performed using DESeq2 package.

fOTUs assigned to *Sporobolomyces* and *Emericellopsis* were the most highly enriched genera in the *cyp79B2cyp79B3* and *tu3* mutants (Fig 6A; **S10 Table in** S1 File). In addition, the fungal family Ceratobasidiaceae was strongly enriched in *tt3*, *tt5*, *tt7_7* and *pap1_D*. A blast analysis of reads assigned this taxon to *Waitea circinata/Rhizoctonia spp*. These strong enrichment of the family Ceratobasidiaceae in *tt3*, *tt5*, *tt7_7* and *pap1_D* compared with parental lines could suggest an antagonistic effect of FLVs on fungal members of this taxa. We also observed differential abundances of notable antagonistic fungi such as *Clonostachys rosea* in *tt3*, *tt5* and *pap1_D*, and the genus *Trichoderma* in *tt5* and *tt7_7* (Fig 6B; **S11 Table in** S1 File). Similarly, in the DSM mutants, *Sporobolomyces* was significantly enriched in *dde2*, *fad3_2* and *fad7_1fad8_1*, while *Emericellopsis* was highly enriched in *dde2* and *etr1_3*. Conversely, *aba3_2* was strongly depleted in *Emericellopsis and Trichoderma* while the ethylene mutants *etr1_3* and *35S*::*ERF* had increased abundances of *Saitozyma* (Fig 6C; **S12 Table in** S1 File).

## Discussion

### GLSs have notable effects on the host root-associated microbiome

Our results revealed notable effects of glucosinolates (GLS) mutants on relative abundances, and alpha- and beta-diversities of microbial communities, suggesting that specific GLS metabolites affect the composition of the root microbiome. The separation of GLS mutants from the parental line in the PCoA plots suggests differential effects of the GLS mutations on the Arabidopsis microbiome, thus supporting previous studies [47]. Specifically, *tu3* that carries a gene disruption upstream of the aliphatic GLS pathway, had the strongest effect on both bacterial and fungal communities. Aliphatic GLS and their hydrolysis products have been reported to have higher effects on microorganisms compared to indole glucosinolates (iGLS) [48]. The toxicity of aliphatic GLS towards microorganisms is attributed to the complex degradation products isothiocyanates, thiocyanates, oxazolidinethiones and nitriles that are produced from the enzymatic cleavage of glucosinolates by myrosinase [49].

The strong effect of *tu3* also confirmed that gene disruptions at the initial steps of a biosynthetic pathway generally have more pronounced effects on the host-associated microbiota [21]. However, other mutants including *cyp79B3* and the double mutant *cyp79B2cyp79B3* in the same indole GLS pathways or the *gsm1_1* in the aliphatic GLS pathways, with upstream gene disruptions only had minor, but significant, effects on the microbiome. These results suggest a differential regulatory role of metabolic genes and their effects on host associated microbiota. Comparatively, the variations in glucosinolates profiles could cause the differential effects of mutants *tu3* and *gsm1-1* on microbial communities. The *tu3* produce glucosinolates that are deficient in *gsm1-1* (that is, aliphatic glucosinolates with butyl, pentyl, or hexyl core groups) but lacks aliphatic glucosinolates with heptyl and octyl core groups [3, 12]. Brader *et al*. [50] found differences in the induction of the CYP79B2 and CYP79B3 genes upon treatment with culture filtrates of the bacterium *Erwinia carotovora*. Hence, it is possible that unknown enzymatic and pleiotropic activities of the mutated genes could contribute to the observed differences of microbial communities. Furthermore, Ludwig-Müller *et al*. [51] reported that several TU mutants, having different contents of GLS intermediate products, developed varying degrees of clubroot disease symptoms caused by *Plasmodiophora brassicae*. Together, these suggest distinct gene functions in GLS pathways which possibly underline mechanistic processes in microbiome assembly.

GLSs had distinct effects on specific microbial groups as confirmed by the identification of a range of bacterial and fungal taxa responding to the different GLS mutations. The increased abundance of individual bacterial genera such as *Azospirillum* and *Fluviicola* as well as the fungal genera *Sporobolomyces* and *Emericellopsis* in several of the GLS mutants further confirmed the selective effects of metabolites on individual microbial taxa and thus corroborates previous studies [21, 23]. For instance, soils amended with isothiocyanates (allyl, butyl, phenyl, and benzyl ITC) were reported to affect fungal communities more dramatically than bacterial communities [52]. The authors observed changes in community composition including increased *Humicola* abundance in allyl ITC and *Mortierella* abundance in butyl ITC amended soils, while the bacterial phylum Firmicutes temporally increased in response to amendment with allyl ITC [52]. Plant metabolic compounds with antimicrobial properties including GLS are known to be part of the boundary layers of the root rhizoplane that modulate root microbiome assemblage [53]. Other genera such as *Nocardioides* and the plant beneficial taxa *Streptomyces* and *Flavobacterium* were also enriched in most of the GLS mutants. *Azospirillum* contains several beneficial species, widely known for their plant growth promoting traits including nitrogen fixation and synthesis of phytohormones and other compounds required for both biotic and abiotic stress tolerance [54]. The yeast *Sporobolomyces*, that was enriched in the GLS lines and the other mutants, is an abundant member of the plant mycobiome [55, 56] and is antagonistic against pathogens [57]. Also, the strongly enriched genus *Emericellopsis* in both the indole GLS mutants *cyp79B2cyp79B3*, *myb51* and aliphatic GLS lines *gsm1_1* and *tu3* is known to possess biocontrol traits via the antimicrobial compound emericellipsin A, and have been shown to suppress the pathogen *Aspergillus niger* [58]. In addition, the strong enrichment of *Fusarium* in *pen3_1_NahG* could suggest that both physical and chemical barriers (GLS and SA) affect this genus [59, 60].

### Flavonoids have a higher effect on root-associated fungal communities

Flavonoids (FLVs) are some of the most studied phytochemicals due to their profound role in plant-microbe interactions. Analysis of microbiota from FLV mutants impaired in different steps of the FLV pathway revealed differential effects on bacterial and fungal communities. While the FLV mutations did not affect bacterial alpha diversities significantly, weak but significant effects of the individual FLV mutations were observed on microbial community composition. The FLV mutant *tt7_7* which lacks orthodihydroxy flavonoids (for example quercetin, naringenin, genistein, luteolin, daidzein, and morin), and accumulates pelargonidin rather than the cyanidin found in wild-type plants [61], had significant effects on both bacterial and fungal communities. Differential responses of pelargonidin and cyanidin to both fungi and bacteria have been reported earlier [62]. The orthodihydroxy flavonoids have been shown to mediate plant-microbe interactions, especially in nodule formation and in enhancing arbuscular mycorrhizal colonization, and by inhibiting bacterial and fungal pathogens [63, 64]. Hence, the disruption of the synthesis of these FLVs would likely affect microbial communities and the strong enrichment of the bacterial genera *Flavobacterium* and *Rhodanobacter* in *tt7_7* could suggest a modulating role of orthodihydroxy flavonoids on bacterial communities. Similarly, the *pap1_D* which accumulates anthocyanin pigments, (mainly cyanins) and other secondary metabolites [65, 66] significantly affected only the fungal communities.

The *tt3* mutant which accumulates high concentrations of both kaempferol and quercetin [13] had a more profound effect on fungal communities compared to the bacterial communities. Both kaempferol and quercetin are highly secreted in Arabidopsis [67], and their accumulation were likely to affect microbial communities. Quercetin enhances mycorrhizal-plant symbiosis by stimulating host penetration and hyphal growth [5], while kaempferol inhibits germination of pathogenic fungal spores [68]. However, Vikram *et al*. [69] reported that kaempferol and quercetin disrupts quorum sensing and biofilm formation in bacterial communities. Schultz *et al*. [70] found higher relative abundances of Proteobacteria in a quercetin-treated soil compared with non-treated soil. Moreover, alpha diversity indices were observed to significant decline after quercetin treatment [70]. Yu *et al*., [71] showed that flavones lead to enrichment of the plant beneficial Oxalobacteraceae in the rhizosphere of maize. Guenoune *et al*. [72] reported antifungal effects of the FLV maackiain against the fungal pathogen *Rhizoctonia solani*. Moreover, the enrichment of some members of the order Pleosporales (genus *Paraphaeosphaeria*) in *tt3* and *tt5*, *Westerdykella* in *tt5* and *tt7_7* or depletion of *Alternaria* in *tt7_7*, suggests differential effects of FLVs on members of this order. The species *Holtermanniella takashimae*, which was enriched in *tt3* and *tt5*, was reported to be negatively co-occurring with *Fusarium* species that were pathogenic in wheat [73].

Although *tt5* (impaired in naringenin chalcone) did not affect microbial community composition significantly, naringenin chalcone inhibits spore germination of plant pathogens [68]. In addition, Vandeputte *et al*. [68, 74] demonstrated that plant produced naringenin and catechin is important in reducing the production of quorum sensing-controlled virulence factors in *Pseudomonas aeruginosa* PAO1. The higher number of differentially abundant fungal taxa compared to bacterial taxa, also point to a higher effect of FLVs on fungi, thus supporting the profound role of FLVs on fungi [75].

### Defense signalling mutations have complex effects on microbial taxa

Defense signaling molecules (DSMs) including JA, ABA, SA, FAD and the gaseous molecule ET are well known for their role in mediating plant-microbe interactions. We found that DSM mutants distinctively affected microbial relative abundances and alpha- and beta diversity, thus confirming previous studies [24, 76, 77]. Both *etr1_3* (ET insensitive) and *35S*::*ERF* (high ET synthesis) displayed noticeable differences in bacterial and fungal relative abundances and diversity, and thus supports previous studies [78, 79]. The distinct effect of *etr1_3* and *35S*::*ERF* could be caused by their differential activation of ethylene. The *etr1_3* has reduced ethylene binding activity while *35S*::*ERF* encodes a transcription factor that regulates plant-microbe interactions, as well as integration of signaling pathways to activate ethylene and jasmonate-dependent responses to pathogens [80, 81]. Using a sterile system with artificially constructed bacterial community, Bodenhausen *et al*. [78] showed that the ethylene-insensitive mutant *ein2* assemble distinct bacterial community compared with the parental line, with a noticeable enrichment of the genus *Variovorax*. Comparably, our study revealed differential enrichment of the genus *Variovorax*, suggesting a selective effect of *etr1_3* on this genus. ABA is an essential molecule in modulating abiotic stress (e.g drought stress and salinity stress), as well as overall plant associated microbial communities [82]. We found that the ABA deficient mutant *aba3_2* affected both bacterial and fungal communities, but surprisingly, only a few bacterial taxa at the genus level, including *Bradyrhizhobium* and *Pseudarthrobacter* were slightly enriched, whereas several fungal genera were enriched. These results indicate a higher antagonistic effect of ABA on fungal communities. In another study, exogenous application of ABA was found to change community composition as well as enrich the genera *Massilia*, *Cellvibrio*, *Limnobacter* [83]. Also, the JA mutant *dde2* (impaired in JA biosynthesis) significantly affected bacterial fungal community composition, with a strong enrichment of bacterial taxa corroborating previous studies [77, 78]. In addition, FAD is pivotal in the phytohormone signalling network by modulating both the SA [84] and JA pathways [85], and its role in mediating plant-microbe interactions has been reported [18, 24]. Likewise, our study revealed distinct effects of FAD mutants on microbial community structures, as both *Azospirillum and Sporobolomyces* were strongly enriched in *fad3_2* and *fad7_1fad8_1*, as was also observed in a previous study in which the Arabidopsis triple mutant *fad3fad7fad8* was enriched in several species within Alpha- and Gammaproteobacteria [24]. Differential effects of FAD genes on microbial taxa have been reported, for instance, while the transcription of the FAD3 gene was shown to be unresponsive upon inoculation of the bacterial pathogen *Xanthomonas campestris* [84], the FAD7 gene was induced by fungal effectors [86]. The distinct enrichment of a number of bacterial genera in the different DSM mutants is indicative of the selective effects of DSMs in shaping the Arabidopsis root microbiome. However, the resident microbial community can interfere with the plant-hormonal pathways [87]. For example, Finkel *et al*. [88] demonstrated that the bacterial genus *Variovorax* utilizes an auxin-degradation operon to alter plant-hormone balances, enabling it to reverse the severe inhibition of root growth that was induced by a wide diversity of bacterial strains. Thus, the analysis of the effect of plant DSMs on microbiomes should be done with caution.

The increasing interest in studying plant metabolites will enable us to better quantify plant host effects on associated microbial communities. However, it is currently challenging to quantify specific effects and mechanisms of important metabolites on plant microbiomes due to methodological limitations. For example, when using mutants, pleiotropic effects arising from gene disruptions in both metabolic and hormonal pathways, makes it impossible to account for individual effects of targeted compounds on microbial community structures. Moreover, because immune signaling activation is complex due to hormonal crosstalk mechanisms it is difficult to quantify the effects of individual hormones on microbial communities. We therefore suggest that, in future studies, detailed analyses should include mutants having complete abolishment of interactive pathways and be complemented with other omics analysis techniques. Furthermore, the host-associated microbiota can alter metabolite synthesis and are also capable of producing several phytohormones [89] and it is therefore important to adopt experimental approaches that will be able to strictly account for plant-derived compounds and their impact on the plant microbiome.

## Conclusion

Arabidopsis mutants carrying gene disruptions in pathways of the plant secondary metabolites GLS and FLV, or the signalling molecules SA, ABA, ET, or FAD, assembled distinct microbiomes compared to their parental lines. Most earlier studies on the effects of disruption of metabolic pathway have only considered bacterial communities. In this study, we demonstrated dramatic effects of such mutations also on fungal communities. We found distinct relative abundances and diversities of bacterial and fungal taxa in the mutants. Differential analysis at OTU level revealed significantly affected taxa between the mutants and parental lines. Also, the bacterial and fungal genera were mostly enriched than depleted in mutants, except for flavonoid mutations on fungi communities. These results strongly support the perception that many synthesized plant secondary metabolites and DSMs regulate the assembly of the plant root microbiome. However, the interconnectedness in metabolic and signalling pathways presents a high complexity, and thus, mutant lines with several mutations with the possible elimination of overlapping defense-signalling functions are suggested for further studies. The present screening study revealed significant gene-microbiota links, and thus serve as an important resource for in-depth plant-omics analysis in the future.

## Supporting information

S1 File (PDF)

## References

[1] Colaianni NR, Parys K, Lee HS, Conway JM, Kim NH, Edelbacher N, et al. A complex immune response to flagellin epitope variation in commensal communities. Cell Host Microbe 2021;29(4):635–649.e9. doi: 10.1016/j.chom.2021.02.006 33713602

[2] Erb M, Kliebenstein DJ. Plant secondary metabolites as defenses, regulators, and primary metabolites: The blurred functional trichotomy. Plant Physiol. 2020;184(1):39–52. doi: 10.1104/pp.20.00433 32636341 PMC7479915

[3] Tierens KFMJ, Thomma BPHJ, Brouwer M, Schmidt J, Kistner K, Porzel A, et al. Study of the role of antimicrobial glucosinolate-derived isothiocyanates in resistance of Arabidopsis to microbial pathogens. Plant Physiol. 2001;125(4):1688–99. doi: 10.1104/pp.125.4.1688 11299350 PMC88826

[4] Angus JF, Gardner PA, Kirkegaard JA, Desmarchelier JM. Biofumigation: Isothiocyanates released from brassica roots inhibit growth of the take-all fungus. Plant Soil. 1994;162(1):107–12.

[5] Cesco S, Mimmo T, Tonon G, Tomasi N, Pinton R, Terzano R, et al. Plant-borne flavonoids released into the rhizosphere: Impact on soil bio-activities related to plant nutrition. A review. Biol Fertil Soils. 2012;48(2):123–49.

[6] Buer CS, Imin N, Djordjevic MA. Flavonoids: New roles for old molecules. J Integr Plant Biol. 2010;52(1):98–111. doi: 10.1111/j.1744-7909.2010.00905.x 20074144

[7] Pieterse CMJ, Leon-Reyes A, Van Der Ent S, Van Wees SCM. Networking by small-molecule hormones in plant immunity. Nat Chem Biol. 2009;5(5):308–16. doi: 10.1038/nchembio.164 19377457

[8] Pieterse CMJ, Van der Does D, Zamioudis C, Leon-Reyes A, Van Wees SCM. Hormonal Modulation of Plant Immunity. Annu Rev Cell Dev Biol 2012;28(1):489–521. doi: 10.1146/annurev-cellbio-092910-154055 22559264

[9] Teixeira PJPL, Colaianni NR, Law TF, Conway JM, Gilbert S, Li H, et al. Specific modulation of the root immune system by a community of commensal bacteria. Proc Natl Acad Sci. 2021;118(16). doi: 10.1073/pnas.2100678118 33879573 PMC8072228

[10] Stringlis IA, Pieterse CMJ. Evolutionary “hide and seek” between bacterial flagellin and the plant immune system. Cell Host Microbe. 2021;29(4):548–50. doi: 10.1016/j.chom.2021.03.010 33857418

[11] Halkier BA, Gershenzon J. Biology and biochemistry of glucosinolates. Annu Rev Plant Biol 2006;57:303–33. doi: 10.1146/annurev.arplant.57.032905.105228 16669764

[12] Haughn GW, Davin L, Giblin M, Underhill EW. Biochemical genetics of plant secondary metabolites in *Arabidopsis thaliana*. Plant Physiol 1991;217–26. doi: 10.1104/pp.97.1.217 16668374 PMC1080987

[13] Peer WA, Brown DE, Tague BW, Muday GK, Taiz L, Murphy AS. Flavonoid accumulation patterns of transparent testa mutants of Arabidopsis. Plant Physiol 2001;126(2):536–48. doi: 10.1104/pp.126.2.536 11402185 PMC111147

[14] Saito K, Yonekura-Sakakibara K, Nakabayashi R, Higashi Y, Yamazaki M, Tohge T, et al. The flavonoid biosynthetic pathway in Arabidopsis: Structural and genetic diversity. Plant Physiol Biochem. 2013;72:21–34. doi: 10.1016/j.plaphy.2013.02.001 23473981

[15] Weston LA, Mathesius U. Flavonoids: Their structure, biosynthesis and role in the rhizosphere, including allelopathy. J Chem Ecol. 2013;39(2):283–97. doi: 10.1007/s10886-013-0248-5 23397456

[16] Staswick PE, Yuen GY, Lehman CC. Jasmonate signaling mutants of Arabidopsis are susceptible to the soil fungus *Pythium irregulare*. Plant J. 1998;15(6):747–54. doi: 10.1046/j.1365-313x.1998.00265.x 9807813

[17] Soliman S, El-Keblawy A, Mosa K, Helmy M, Wani S. Understanding the phytohormones biosynthetic pathways for developing engineered environmental stress-tolerant crops. In: Biotechnologies of Crop Improvement, Volume 2: Transgenic Approaches. 2018. p. 417–50.

[18] Kachroo P, Shanklin J, Shah J, Whittle EJ, Klessig DF. A fatty acid desaturase modulates the activation of defense signaling pathways in plants. Proc Natl Acad Sci. 2002;98(16):9448–53. 10.1073/pnas.151258398 PMC55441 11481500

[19] Badri D V., Quintana N, El Kassis EG, Kim HK, Choi YH, Sugiyama A, et al. An ABC transporter mutation alters root exudation of phytochemicals that provoke an overhaul of natural soil microbiota. Plant Physiol 2009;151(4):2006–17. doi: 10.1104/pp.109.147462 19854857 PMC2785968

[20] Bressan M, Roncato M-A, Bellvert F, Comte G, el Haichar F Z, Achouak W, et al. Exogenous glucosinolate produced by *Arabidopsis thaliana* has an impact on microbes in the rhizosphere and plant roots. ISME J. 2009;3(11):1243–57. doi: 10.1038/ismej.2009.68 19554039

[21] Kudjordjie EN, Sapkota R, Steffensen SK, Fomsgaard IS, Nicolaisen M. Maize synthesized benzoxazinoids affect the host associated microbiome. Microbiome. 2019;1–17. 30975184 10.1186/s40168-019-0677-7 PMC6460791

[22] Stringlis IA, Yu K, Feussner K, de Jonge R, Van Bentum S, Van Verk MC, et al. MYB72-dependent coumarin exudation shapes root microbiome assembly to promote plant health. Proc Natl Acad Sci. 2018;115(22):E5213–22. doi: 10.1073/pnas.1722335115 29686086 PMC5984513

[23] Voges MJEEE, Bai Y, Schulze-Lefert P, Sattely ES. Plant-derived coumarins shape the composition of an Arabidopsis synthetic root microbiome. Proc Natl Acad Sci. 2019;116(25):12558–12565. doi: 10.1073/pnas.1820691116 31152139 PMC6589675

[24] Kniskern JM, Traw MB, Bergelson J. Salicylic acid and jasmonic acid signaling defense pathways reduce natural bacterial diversity on *Arabidopsis thaliana*. MPMI. 2007;20(12):1512–22. doi: 10.1094/MPMI-20-12-1512 17990959

[25] Zapata L, Ding J, Willing EM, Hartwig B, Bezdan D, Jiao WB, et al. Chromosome-level assembly of *Arabidopsis thaliana* Ler reveals the extent of translocation and inversion polymorphisms. Proc Natl Acad Sci. 2016;113(28):E4052–60. doi: 10.1073/pnas.1607532113 27354520 PMC4948326

[26] Mönchgesang S, Strehmel N, Schmidt S, Westphal L, Taruttis F, Müller E, et al. Natural variation of root exudates in *Arabidopsis thaliana*-linking metabolomic and genomic data. Sci Rep 2016;6:29033. doi: 10.1038/srep29033 27363486 PMC4929559

[27] Cajero Sánchez W, García-Ponce B, Sánchez M de la P, Álvarez-Buylla ER, Garay-Arroyo A. Identifying the transition to the maturation zone in three ecotypes of *Arabidopsis thaliana* roots. Commun Integr Biol. 2018;11(1). doi: 10.1080/19420889.2017.1395993 29497470 PMC5824961

[28] Price CG, Knee EM, Miller JA, Shin Di, Mann J, Crist DK, et al. Following Phenotypes: An Exploration of mendelian genetics using Arabidopsis plants. Am Biol Teach. 2018;80(4):291–300.

[29] Diener AC, Ausubel FM. RESISTANCE TO FUSARIUM OXYSPORUM 1, a dominant Arabidopsis disease-resistance gene, is not race specific. Genetics. 2005;171(1):305–21. doi: 10.1534/genetics.105.042218 15965251 PMC1456520

[30] Reiss A, Fomsgaard IS, Mathiassen SK, Kudsk P. Silicone tube microextraction for repeated sampling of benzoxazinoids in the root zone and analysis by HPLC /MS-MS. J Allelochem Interact. 2018;2018:27–37.

[31] Ihrmark K, Bödeker ITM, Cruz-Martinez K, Friberg H, Kubartova A, Schenck J, et al. New primers to amplify the fungal ITS2 region—evaluation by 454-sequencing of artificial and natural communities. FEMS Microbiol Ecol. 2012;82(3):666–77. doi: 10.1111/j.1574-6941.2012.01437.x 22738186

[32] Klindworth A, Pruesse E, Schweer T, Peplies J, Quast C, Horn M, et al. Evaluation of general 16S ribosomal RNA gene PCR primers for classical and next-generation sequencing-based diversity studies. Nucleic Acids Res. 2013 Jan;41(1):e1. doi: 10.1093/nar/gks808 22933715 PMC3592464

[33] Rognes T, Flouri T, Nichols B, Quince C, Mahé F. VSEARCH: a versatile open source tool for metagenomics. PeerJ. 2016;4:e2584. doi: 10.7717/peerj.2584 27781170 PMC5075697

[34] Caporaso JG, Kuczynski J, Stombaugh J, Bittinger K, Bushman FD, Costello EK, et al. QIIME allows analysis of high- throughput community sequencing data. Nature Methods. 2010;7(5):335–6. doi: 10.1038/nmeth.f.303 20383131 PMC3156573

[35] Quast C, Pruesse E, Yilmaz P, Gerken J, Schweer T, Yarza P, et al. The SILVA ribosomal RNA gene database project: Improved data processing and web-based tools. Nucleic Acids Res. 2013;41:590–6. doi: 10.1093/nar/gks1219 23193283 PMC3531112

[36] Zinger L, Bonin A, Alsos IG, Bálint M, Bik H, Boyer F, et al. DNA metabarcoding—Need for robust experimental designs to draw sound ecological conclusions. Mol Ecol. 2019;28(8):1857–62. doi: 10.1111/mec.15060 31033079

[37] R Core Team. R: A language and environment for statistical computing. 2017.

[38] Oksanen AJ, Blanchet FG, Friendly M, Kindt R, Legendre P, Mcglinn D, et al. Package ‘ vegan’ 2019.

[39] McMurdie PJ, Holmes S, Kindt R, Legendre P, O’Hara R. phyloseq: An R package for reproducible interactive analysis and graphics of microbiome census data. Watson M, editor. PLoS One. 2013 Apr;8(4):e61217. doi: 10.1371/journal.pone.0061217 23630581 PMC3632530

[40] Parks DH, Tyson GW, Hugenholtz P, Beiko RG. STAMP: Statistical analysis of taxonomic and functional profiles. Bioinformatics. 2014;30(21):3123–4. doi: 10.1093/bioinformatics/btu494 25061070 PMC4609014

[41] Parks DH, Beiko RG. Identifying biologically relevant differences between metagenomic communities. Bioinformatics. 2010;26(6):715–21. doi: 10.1093/bioinformatics/btq041 20130030

[42] Wang Y, Naumann U, Wright ST, Warton DI. Mvabund- an R package for model-based analysis of multivariate abundance data. Methods Ecol Evol. 2012;3(3):471–4.

[43] Love MI, Huber W, Anders S. Moderated estimation of fold change and dispersion for RNA-seq data with DESeq2. Genome Biol. 2014;15(12):1–21. doi: 10.1186/s13059-014-0550-8 25516281 PMC4302049

[44] Sønderby IE, Geu-Flores F, Halkier BA. Biosynthesis of glucosinolates—gene discovery and beyond. Trends Plant Sci. 2010;15(5):283–90. doi: 10.1016/j.tplants.2010.02.005 20303821

[45] Frerigmann H, Gigolashvili T. MYB34, MYB51, and MYB122 distinctly regulate indolic glucosinolate biosynthesis in *Arabidopsis thaliana*. Mol Plant. 2014;7(5):814–28. doi: 10.1093/mp/ssu004 24431192

[46] Stein M, Dittgen J, Sanchez-Rodrıguez C, Hou B-H, Molina A, Schulze-Lefert P, et al. Arabidopsis PEN3/PDR8, an ATP binding cassette transporter, contributes to nonhost resistance to inappropriate pathogens that enter by direct penetration. Plant cell. 2006;18:731–746. doi: 10.1105/tpc.105.038372 16473969 PMC1383646

[47] Micallef S, Colón-Carmona A. Genetic and developmental control of rhizosphere bacterial communities. Mol Microb Ecol Rhizosph. 2013;1:257–63.

[48] Kirkegaard JA, Sarwar M. Biofumigation potential of brassicas I. Variation in glucosinolate profiles of diverse field-grown brassicas. Plant Soil. 1998;71–89.

[49] Burel C, Boujard T, Kaushik SJ, Boeuf G, Mol KA, Van Der Geyten S, et al. Effects of rapeseed meal-glucosinolates on thyroid metabolism and feed utilization in rainbow trout. Gen Comp Endocrinol. 2001;124(3):343–58. doi: 10.1006/gcen.2001.7723 11742518

[50] Brader G, Tas E, Palva ET. Jasmonate-dependent induction of indole glucosinolates in arabidopsis by culture filtrates of the nonspecific pathogen *Erwinia carotovora*. Plant Physiol. 2001;126(2):849–60. doi: 10.1104/pp.126.2.849 11402212 PMC111174

[51] Ludwig-Müller J, Pieper K, Ruppel M, Cohen JD, Epstein E, Kiddle G, et al. Indole glucosinolate and auxin biosynthesis in *Arabidopsis thaliana* (L.) Heynh. glucosinolate mutants and the development of clubroot disease. Planta. 1999;208(3):409–19. doi: 10.1007/s004250050576 10384731

[52] Hu P, Hollister EB, Somenahally AC, Hons FM, Gentry TJ. Soil bacterial and fungal communities respond differently to various isothiocyanates added for biofumigation. *Front Microbiol* 2014; 5: 1–9. 25709600 10.3389/fmicb.2014.00729 PMC4288022

[53] van der Heijden MGA, Schlaeppi K. Root surface as a frontier for plant microbiome research: Proc Natl Acad Sci. 2015;112(8):2299–300. doi: 10.1073/pnas.1500709112 25713090 PMC4345591

[54] Fukami J, Cerezini P, Hungria M. Azospirillum: Benefits that go far beyond biological nitrogen fixation. AMB Express. 2018;8(1):1–12. 29728787 10.1186/s13568-018-0608-1 PMC5935603

[55] Sapkota R, Knorr K, Jørgensen LN, O Hanlon K a, Nicolaisen M. Host genotype is an important determinant of the cereal phyllosphere mycobiome. New Phytol. 2015;207(4):1134–44. doi: 10.1111/nph.13418 25898906

[56] Sapkota R, Jørgensen LN, Nicolaisen M. Spatiotemporal variation and networks in the mycobiome of the wheat canopy. Front Plant Sci. 2017;8:1357. doi: 10.3389/fpls.2017.01357 28824687 PMC5539183

[57] Wachowska U, Borowska J. Antagonistic yeasts competes for iron with winter wheat stem base pathogensAntagonistische Hefen konkurrieren mit Winterweizenhalmbasis-Pathogenen um Eisen. Gesunde Pflanz. 2014;66(4):141–8.

[58] Rogozhin EA, Sadykova VS, Baranova AA, Vasilchenko AS, Lushpa VA, Mineev KS, et al. A novel lipopeptaibol emericellipsin a with antimicrobial and antitumor activity produced by the extremophilic fungus emericellopsis alkalina. Molecules. 2018;23(11). doi: 10.3390/molecules23112785 30373232 PMC6278523

[59] Edgar CI, McGrath KC, Dombrecht B, Manners JM, Maclean DC, Schenk PM, et al. Salicylic acid mediates resistance to the vascular wilt pathogen *Fusarium oxysporum* in the model host Arabidopsis thaliana. Australas Plant Pathol. 2006;35(6):581–91.

[60] Berrocal-Lobo M, Molina A. Arabidopsis defense response against *Fusarium oxysporum*. Trends Plant Sci. 2008;13(3):145–50. doi: 10.1016/j.tplants.2007.12.004 18289920

[61] Koornneef M, Luiten W, de Vlaming P, Schram AW. A gene controlling flavonoid 3’ hydroxylation in Arabidopsis. Arab Inf Serv. 1982;19(1980):113–5.

[62] Lingua G, Bona E, Manassero P, Marsano F, Todeschini V, Cantamessa S, et al. Arbuscular mycorrhizal fungi and plant growth-promoting pseudomonads increases anthocyanin concentration in strawberry fruits (Fragaria x ananassa var. Selva) in conditions of reduced fertilization. Int J Mol Sci. 2013;14(8):16207–25. doi: 10.3390/ijms140816207 23924942 PMC3759907

[63] Shah A, Smith DL. Flavonoids in agriculture: Chemistry and roles in, biotic and abiotic stress responses, and microbial associations. Agronomy. 2020;10(8).

[64] Sugiyama A, Yazaki K. Flavonoids in plant rhizospheres: Secretion, fate and their effects on biological communication. Plant Biotechnol. 2014;31(5):431–43.

[65] Borevitz JO, Xia Y, Blount J, Dixon RA, Lamb C. Activation tagging identifies a conserved myb regulator of phenylpropanoid biosynthesis. Plant Cell. 2000;12(12):2383. doi: 10.1105/tpc.12.12.2383 11148285 PMC102225

[66] Shi MZ, Xie DY. Features of anthocyanin biosynthesis in pap1-D and wild-type *Arabidopsis thaliana* plants grown in different light intensity and culture media conditions. Planta. 2010;231(6):1385–400. doi: 10.1007/s00425-010-1142-9 20309578

[67] Buer CS, Djordjevic MA. Architectural phenotypes in the transparent testa mutants of *Arabidopsis thaliana*. J Exp Bot. 2009;60(3):751–63. doi: 10.1093/jxb/ern323 19129166 PMC2652062

[68] Vandeputte OM, Kiendrebeogo M, Rajaonson S, Diallo B, Mol A, Jaziri M El, et al. Identification of catechin as one of the flavonoids from *Combretum albiflorum* bark extract that reduces the production of quorum-sensing-controlled virulence factors in *Pseudomonas aeruginosa* PAQ1. Appl Environ Microbiol. 2010;76(1):243–53. doi: 10.1128/AEM.01059-09 19854927 PMC2798626

[69] Vikram A, Jayaprakasha GK, Jesudhasan PR, Pillai SD, Patil BS. Suppression of bacterial cell-cell signalling, biofilm formation and type III secretion system by citrus flavonoids. J Appl Microbiol. 2010;109(2):515–27. doi: 10.1111/j.1365-2672.2010.04677.x 20163489

[70] Schütz V, Frindte K, Cui J, Zhang P, Hacquard S, Schulze-Lefert P, et al. Differential Impact of Plant Secondary Metabolites on the Soil Microbiota. *Front Microbiol* 2021; 12: 1–17. doi: 10.3389/fmicb.2021.666010 34122379 PMC8195599

[71] Yu P, He X, Baer M, Beirinckx S, Tian T, Moya YAT, et al. Plant flavones enrich rhizosphere Oxalobacteraceae to improve maize performance under nitrogen deprivation. *Nat Plants* 2021; 7: 481–499. doi: 10.1038/s41477-021-00897-y 33833418

[72] Guenoune D, Galili S, Phillips DA, Volpin H, Chet I, Okon Y, et al. The defense response elicited by the pathogen *Rhizoctonia solani* is suppressed by colonization of the AM-fungus *Glomus intraradices*. Plant Sci. 2001;160(5):925–32. doi: 10.1016/s0168-9452(01)00329-6 11297789

[73] Rojas EC, Sapkota R, Jensen B, Jørgensen HJL, Henriksson T, Jørgensen LN, et al. Fusarium head blight modifies fungal endophytic communities during infection of wheat spikes. Microb Ecol. 2020;79(2):397–408. doi: 10.1007/s00248-019-01426-3 31448388 PMC7033075

[74] Vandeputte OM, Kiendrebeogo M, Rasamiravaka T, Stévigny C, Duez P, Rajaonson S, et al. The flavanone naringenin reduces the production of quorum sensing-controlled virulence factors in *Pseudomonas aeruginosa* PAO1. Microbiology. 2011;157(7):2120–32. doi: 10.1099/mic.0.049338-0 21546585

[75] Scervino JM, Ponce MA, Erra-Bassells R, Vierheilig H, Ocampo JA, Godeas A. Flavonoids exhibit fungal species and genus specific effects on the presymbiotic growth of Gigaspora and Glomus. Mycol Res. 2005;109(7):789–94. doi: 10.1017/s0953756205002881 16121564

[76] Lebeis SL, Paredes SH, Lundberg DS, Breakfield N, Gehring J, McDonald M, et al. Salicylic acid modulates colonization of the root microbiome by specific bacterial taxa. Science. 2015;349(6250):860–4. doi: 10.1126/science.aaa8764 26184915

[77] Carvalhais LC, Dennis PG, Badri D V, Kidd BN, Vivanco JM, Schenk PM. Linking jasmonic acid signaling, root exudates, and rhizosphere microbiomes. Mol Plant Microbe Interact 2015;28(9):1049–58. doi: 10.1094/MPMI-01-15-0016-R 26035128

[78] Bodenhausen N, Bortfeld-Miller M, Ackermann M, Vorholt JA. A Synthetic community approach reveals plant genotypes affecting the phyllosphere microbiota. PLoS Genet. 2017;10(4). 10.1371/journal.pgen.1004283 PMC3990490 24743269

[79] Doornbos RF, Geraats BPJ, Kuramae EE, Van Loon LC, Bakker P a HM. Effects of jasmonic acid, ethylene, and salicylic acid signaling on the rhizosphere bacterial community of *Arabidopsis thaliana*. Mol Plant Microbe Interact. 2011;24(4):395–407. doi: 10.1094/MPMI-05-10-0115 21171889

[80] Negi S, Ivanchenko MG, Muday GK. Ethylene regulates lateral root formation and auxin transport in *Arabidopsis thaliana*. Plant J. 2008;55(2):175–87. doi: 10.1111/j.1365-313X.2008.03495.x 18363780 PMC2635504

[81] Solano R, Stepanova A, Chao Q, Ecker JR. Nuclear events in ethylene signaling: A transcriptional cascade mediated by ETHYLENE-INSENSITIVE3 and ETHYLENE-RESPONSE-FACTOR1. Genes Dev. 1998;12(23):3703–14. doi: 10.1101/gad.12.23.3703 9851977 PMC317251

[82] Jones P, Garcia BJ, Furches A, Tuskan GA, Jacobson D. Plant host-associated mechanisms for microbial selection. Front Plant Sci. 2019;10:1–14. 31333701 10.3389/fpls.2019.00862 PMC6618679

[83] Carvalhais LC, Dennis PG, Schenk PM. Plant defence inducers rapidly influence the diversity of bacterial communities in a potting mix. Appl Soil Ecol. 2014;84:1–5.

[84] Buell CR, Somerville SC. Expression of defense-related and putative signaling genes during tolerant and susceptible interactions of Arabidopsis with *Xanthomonas campestris pv*. *campestris*. MPMI. 1995. p. 435–43.

[85] Kirsch C, Takamiya-Wik M, Reinold S, Hahlbrock K, Somssich IE. Rapid, transient, and highly localized induction of plastidial ω-3 fatty acid desaturase mRNA at fungal infection sites in *Petroselinum crispum*. Proc Natl Acad Sci. 1997;94(5):2079–84. doi: 10.1073/pnas.94.5.2079 9050908 PMC20046

[86] Avila CA, Arévalo-Soliz LM, Jia L, Navarre DA, Chen Z, Howe GA, et al. Loss of function of FATTY ACID DESATURASE7 in tomato enhances basal aphid resistance in a salicylate-dependent manner. Plant Physiol. 2012;158(4):2028–41. doi: 10.1104/pp.111.191262 22291202 PMC3320204

[87] Compant S, Samad A, Faist H, Sessitsch A. A review on the plant microbiome: Ecology, functions, and emerging trends in microbial application. J Adv Res. 2019;(March). 10.1016/j.jare.2019.03.004 PMC6630030 31341667

[88] Finkel OM, Salas-González I, Castrillo G, Conway JM, Law TF, Teixeira PJPL, et al. A single bacterial genus maintains root growth in a complex microbiome. Nature. 2020;587(7832):103–8. doi: 10.1038/s41586-020-2778-7 32999461 PMC10329457

[89] Trivedi P, Leach JE, Tringe SG, Sa T, Singh BK. Plant–microbiome interactions: from community assembly to plant health. Nat Rev Microbiol. 2020;1–15. 32788714 10.1038/s41579-020-0412-1

