# Supplementary material for: Arabidopsis assemble distinct root-associated microbiomes through the synthesis of an array of defense metabolites
Source: PLoS One. 2021 Oct 26;16(10):e0259171. doi: 10.1371/journal.pone.0259171 (PMC8547673; doi:10.1371/journal.pone.0259171)
Supplement: S1 File — (PDF) [file pone.0259171.s001.pdf]

Supplementary file

Additional file 1

Arabidopsis sculpt distinct root-associated microbiomes through synthesis of secondary metabolites and defense signaling molecules

Enoch Narh Kudjordjie[1], Rumakanta Sapkota[1,#b], Mogens Nicolaisen[1*]

Supplementary Method

Library preparation

Frozen samples were lyophilized for 72 h and ground using sterile steel beads in a Geno/Grinder2000 at 1500 rpm for 3 × 30 s. For DNA extraction 250 mg was weighed and extracted with the PowerLyzer™ PowerSoil® DNA Isolation Kit (Mo Bio Laboratories, Carlsbad, CA, USA), following the manufacturer's instructions. The DNA samples were stored at −20 °C prior to downstream processing.

The 16S and ITS amplicon libraries were generated respectively using the bacterial primers S-D-Bact-0341-b-S-17, 5′-CCTACGGGNGGCWGCAG-3′ and S-D-Bact-0785-a-A-21, 5′-GACTACHVGGGTATCTAATCC-3′ (1) and the fungal primers fITS7, 5′-GTGARTCATCGAATCTTTG-3′ and ITS4, 5′-TCCTCCGCTTATTGATATGC-3′ (2). The primers were extended at the 5`end with barcodes for multiplexed library sequencing (3). Bacterial and fungal primer pairs, respectively, amplified the V3/V4 region of the bacterial 16S rRNA and the fungal internal transcribed spacer 2 (ITS2) region. PCR reactions were performed on GeneAmp PCR System 9700 thermal cycler (Thermo Fisher Scientific). Each 16S PCR was performed in a reaction mixture of 25 µl consisting of 1× PCR reaction buffer, 1.5 mM $MgCl_2$, 0.2 mM dNTPs, 1 µM of each primer, 1 U of GoTaq Flexi polymerase (Promega Corporation, Madison, USA), and 1 µl of DNA template. Thermal cycling conditions are as follows; 94 °C for 5 min, followed by 25 cycles at 94 °C for 30 s, 55 °C for 30 s, 72 °C for 1 min, and a final elongation step at 72 °C for 10 min. The fungal (ITS) libraries were prepared similarly, except that we used an annealing temperature of 57 °C as recommended (1). Dual indexing in combination with internal barcodes was carried out to allow pooling of samples. For indexing, primers including indexing tags were used in a PCR for 10 cycles, with the thermal cycler programs as described

above. In addition to dual indexing, a varying number of nucleotides were added to the forward primer as internal barcodes for combining samples within each index combination [2, 4]. Primer sequences including internal barcodes and the index combinations are described in (5).

The PCR amplicon sizes were confirmed by visualization in a 1.5% agarose gel using SYBR staining, and the PCR products subsequently pooled, precipitated, and re-eluted as described earlier (6). In order to extract the expected size of the amplicons (300–450 bp) and to avoid shorter reads, the pooled DNA was separated on a 1.5% agarose gel and the band of the expected size was extracted using a QIAquick Gel Extraction Kit (Qiagen). The final concentration of the amplicon library was evaluated using a spectrophotometer. Two sequencing libraries, one each for bacteria and fungi were sequenced using the Illumina MiSeq platform at Eurofins MWG (Ebersberg, Germany). All the sequence files were deposited in the NCBI Sequence Read Archive (SRA) under the accession number PRJNA579829.

References

1. Ihrmark K., Bödeker I.T.M., Cruz-Martinez K., Friberg H., Kubartova A., Schenck J., … Lindahl BD. (2012). New primers to amplify the fungal ITS2 region--evaluation by 454-sequencing of artificial and natural communities. *FEMS Microbiology Ecology*, 82, 666–77.
2. Siddique A.B., & Unterseher M. (2016). A cost-effective and efficient strategy for Illumina sequencing of fungal communities: A case study of beech endophytes identified elevation as main explanatory factor for diversity and community composition. *Fungal Ecology*, 20, 175–85.
3. Kudjordjie E.N., Sapkota R., Steffensen S.K., Fomsgaard I.S., & Nicolaisen M. (2019). Maize synthesized benzoxazinoids affect the host associated microbiome. *Microbiome*, 1–17.
4. Wu L., Wen C., Qin Y., Yin H., Tu Q., Van Nostrand J.D., … Zhou J. (2015). Phasing amplicon sequencing on Illumina Miseq for robust environmental microbial community analysis. *BMC Microbiology*, 15(125), 1-12.
5. McMurdie P.J., Holmes S., Kindt R., Legendre P., & O'Hara R. (2013). phyloseq: An R package for reproducible interactive analysis and graphics of microbiome census data. *PLoS One*, 8(4), e61217.
6. Sapkota R., & Nicolaisen M. (2018). Cropping history shapes fungal, oomycete and nematode communities in arable soils and affects cavity spot in carrot. *Agriculture, Ecosystems & Environment*, 257, 120–31.

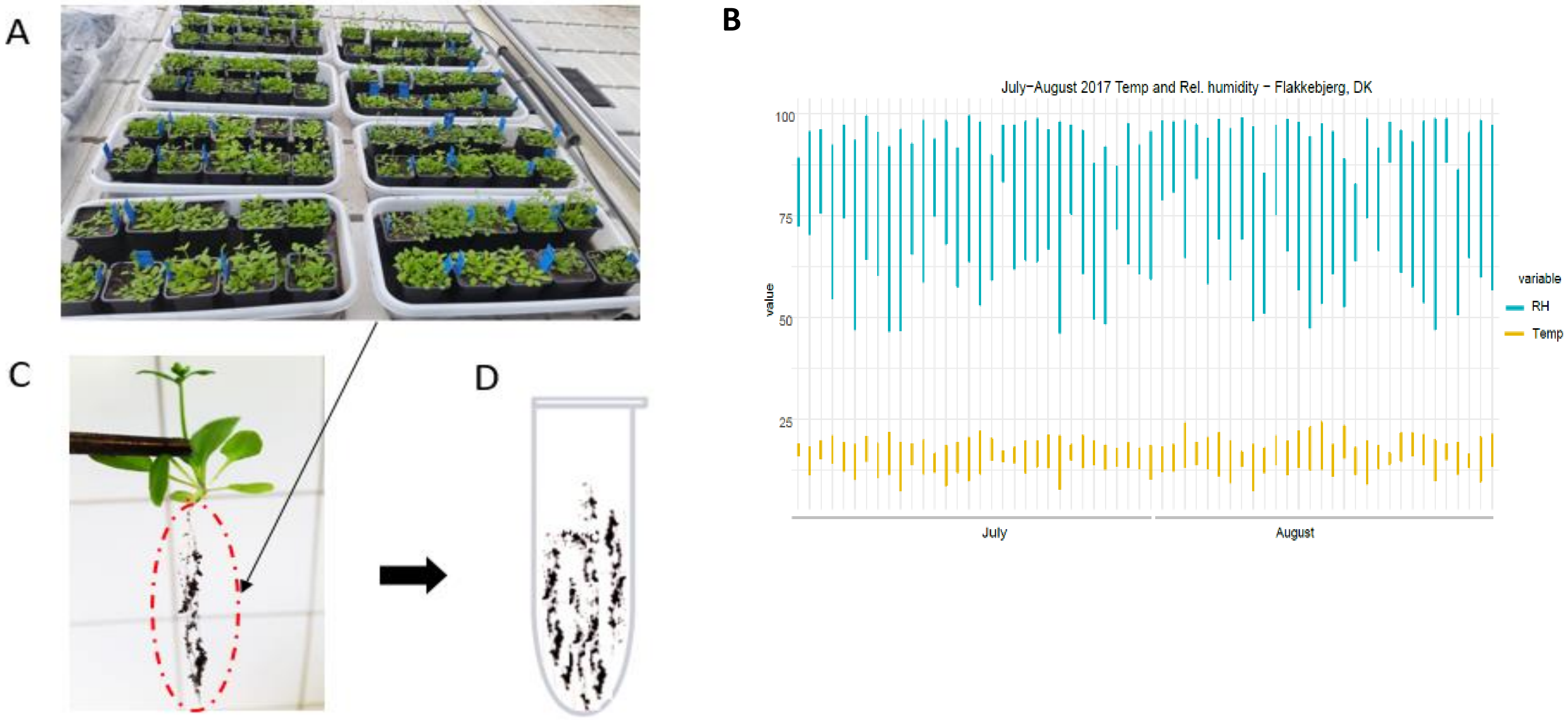

Figure S1. Arabidopsis growth and harvesting scheme. **A**) Arabidopsis growing under greenhouse conditions. **B**) Summer conditions (July-August temperature and relative humidity (RH) for the growth of Arabidopsis in greenhouses. Each bar represent daily readings (at hourly intervals) (Source: DMI Dalmose Station). **C**) Arabidopsis with the roots (circled in red), the part harvested for analysis D) Root samples from individual plants in same pots were pooled into in a collection tube (representing a replicate) and subsequently frozen.

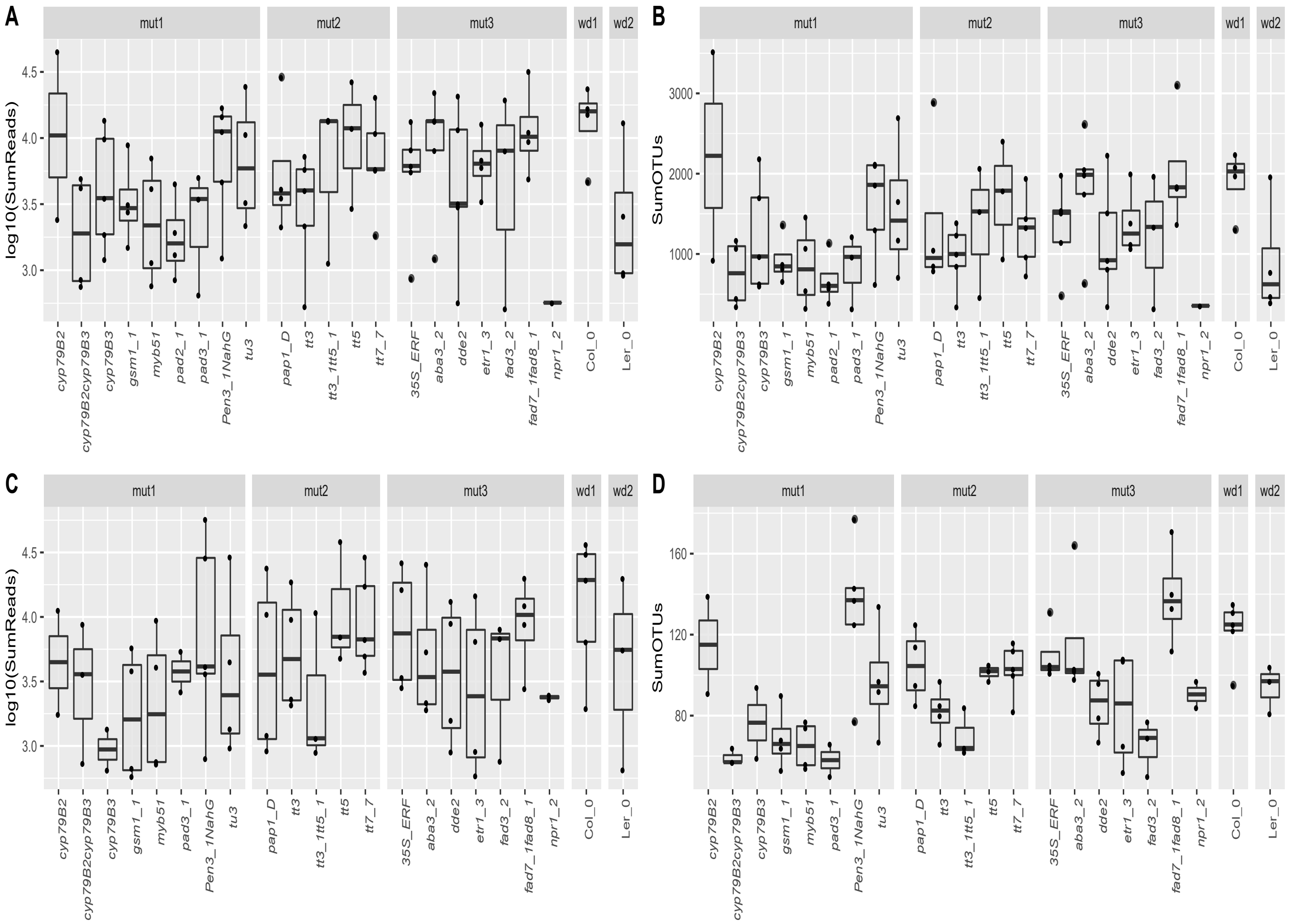

Figure S2. Box plots showing the distribution of number of reads and OTU distribution after pruning in each sample used in this study. Bacterial reads (A) and OTU distribution (B) and the fungal reads (C) and OTU distribution (D) .

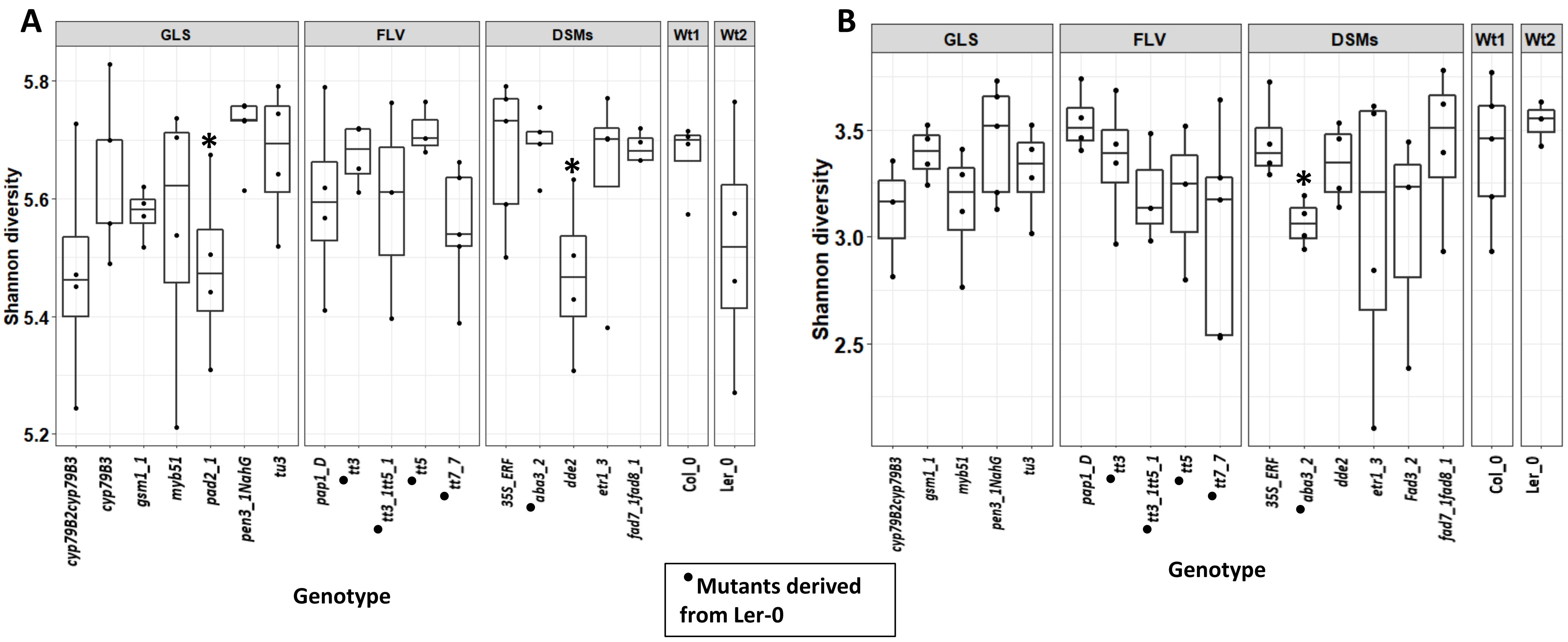

Figure S3. Alpha diversity (Shannon) of bacterial (A) and fungal (B) communities in roots of Arabidopsis secondary metabolite and signalling mutants and their parental lines (Col_0 and Ler-0). Significant differences in alpha diversity in secondary metabolite and signalling mutants and their parental lines are indicated as *, **, and *** for P <0.05, <0.01 and <0.001 respectively. Analysis was performed using the Kruskal- Wallis test.

Supplementary tables

Table S1. List of Arabidopsis accessions used for the experiment

Sample ID	Genotype	Sample type	Description	Source/reference
**GLS**				
22625	Col_0	Wildtype	Reference control	NASC
cyp79B2	*cyp79B2*	mutant	partial IGS synthesis	Prof. J. Bender, Mikkelsen et al., 2000, Hull et al., 2000
cyp79B3	*cyp79B3*	mutant	partial IGS synthesis	Prof. J. Bender, Hull et al., 2000, Zhao et al., 2002
cyp79B279B3	*cyp79B2cyp79B3*	mutant	Total disruption of IGS and camalexin synthesis	Prof. J. Bender, Mikkelsen et al., 2000, Hull et al., 2000
N2226	*gsm1-1 (TU1)*	mutant	Low amino acid substrate required for GLS synthesis. Reduced levels of many aliphatic glucosinolates	NASC, (Haughn et al., 1991)
N2228	*tu3*	mutant	Low amino acid substrate required for GLS synthesis. Deficient in aliphatic GLS with heptyl and octyl core groups.	NASC, Haughn et al., 1991
N56602	*myb51*	mutant	IGS synthesis disruption	NASC, Frerigmann et al., 2014
N67803	*Pen3-1NahG*	mutant	Disruption in both IGS synthesis and SA signaling pathways.<br>PEN3 encodes a pleiotropic drug resistance (PDR) ATP-binding cassette (ABC) transporter involved in the secretion of antimicrobial products, including those resulting from PEN2 activity; a myrosinase (enzyme) that initiates the induction of tryptophan-derived IGS.<br>NahG gene that encodes salicylate hydroxylase (converts SA to catechol) NahG gene suppress expression of PR genes.	NASC, Bednarek et al., 2009; Stein et al., 2006.<br>Yamamoto et al., 1965<br>Delaney et al., 1994.
**Camalexin**				
N3804	*pad2-1*	mutant	Partial camalexin deficient	NASC, Schlaeppi et al., 2008
N3805	*pad3-1*	mutant	Total camalexin deficient	NASC, Glawischnig et al., 2006, Schuhegger et al., 2007
**FLV**				
tt3	*tt3*	mutant	Accumulates excess amounts of quercetin and kaempferol	Peer et al., 2001, Dong et al., 2001
tt5	*tt5*	mutant	Low-level quercetin production	Peer et al., 2001
N2105578	*tt7-7*	mutant	Deficient in flavonoid 3'-hydroxylase activity and lacks orthodihydroxy flavonoids such as quercetin deficient and kaempferol	NASC, Rosso et al., 2003, Appelhagen et al., 2014
N8596	*tt3-1tt5_1*	mutant	Double mutation, disruption of the synthesis of brown pigment	NASC, Peer et al., 2001
N3884	*pap1-D*	mutant	Overexpressed mutant	NASC, Shulz et al., 2016, Borevitz et al., 2000

DSMs				
dde2	*dde2*	mutant	JA deficient	Staswich et al., 1998
N158	*aba3-2*	mutant	ABA deficient	NASC, refs: Leon-Kloosterziel et al. (1996), Schwartz et al. (1997)
N3070	*etr1-3*	mutant	Ethylene responsive	NASC; ref: Woeste et al. (1999), Negi et al. (2008), Binder (2020)
N3801	*npr1-2*	mutant	SA impaired	NASC, ref: Spoel et al. (2003)
N6143	*35S::ERF*	mutant	Ethylene (overexpressed)	NASC, Solano et al., 1998
N8034	*fad3-2*	mutant	Fatty acid desaturase (FAD) deficient (deficient in lineolate desaturase, 18:2; deficient in endoplasmic reticulum lipids; no visible phenotypic effects)	NASC, ref ; Browse et al. (1993)
N8036	*fad7-1fad8-1*	mutant	FAD double mutant (linoleic acid overproducer; linolenic acid underproducer.	NASC, ref: Mcconn et al. (1994)
NW20	Ler-0	Wildtype	Reference control	NASC

The FLV mutants (tt lines) used in this study had a defective proanthocyanidin accumulation resulting in a transparent testa (tt) phenotype characterized by a yellowish or pale brown colour (29). NASC* Nottingham Arabidopsis Stock Centre. * I3AOx (Indole-3-aldoxime),*IGS (indole glucosinolate), *ABA (Abscisic acid), *SA (salicylic acid).

References

1. Appelhagen I, Thiedig K, Nordholt N, Schmidt N, Huep G, Sagasser M, et al. Update on transparent testa mutants from Arabidopsis thaliana: characterisation of new alleles from an isogenic collection. Planta 2014; 240: 955–970.

2. Bednarek P., Piślewska-bednarek M., Svatoš A., Schneider B., Mansurova M., Humphry M., … Schulze-Lefert P. A glucosinolate metabolism pathway in living plant cells mediates broad-spectrum antifungal defense. Science. 2009; 323, 101–6.

3. Borevitz JO, Xia Y, Blount J, Dixon RA, Lamb C: Activation tagging identifies a conserved MYB regulator of phenylpropanoid biosynthesis. Plant Cell. 2000; 12:2383-2394.3.

4. Delaney T.P., Uknes S., Vernooij B., Friedrich L., Weymann K., Negrotto D., … Ryas J. A central role of salicylic acid in plant disease resistance. Science. 1994; 266, 1247–50. Dong XY, Braun EL, Grotewold E: Functional conservation of plant secondary metabolic enzymes revealed by complementation of Arabidopsis flavonoid mutants with maize genes. Plant Physiol. 2001; 127:46-57.

5. Frerigmann H, Gigolashvili T: MYB34, MYB51, and MYB122 distinctly regulate indolic glucosinolate biosynthesis in *Arabidopsis thaliana*. Mol Plant. 2014, 7:814-828.

6. Glawischnig E: The role of cytochrome P450 enzymes in the biosynthesis of camalexin. Biochem. Soc. Trans. 2006; 34:1206-1208.

7. Haughn G.W., Davin L., Giblin M., & Underhill E.W. Biochemical genetics of plant secondary metabolites in *Arabidopsis thaliana*. Plant Physiol. 1991; 217–26.

8. Hull AK, Vij R, Celenza JL: Arabidopsis cytochrome P450s that catalyze the first step of tryptophan-dependent indole-3-acetic acid biosynthesis. Proc Natl Acad Sci. 2000; 97:2379-2384.

9. Mikkelsen MD, Hansen CH, Wittstock U, Halkier BA: Cytochrome P450CYP79B2 from Arabidopsis catalyzes the conversion of tryptophan to indole-3-acetaldoxime, a precursor of indole glucosinolates and indole-3-acetic acid. J. Biol. Chem. 2000; 275:33712-33717.

10. Peer WA, Brown DE, Tague BW, Muday GK, Taiz L, Murphy AS: Flavonoid accumulation patterns of transparent testa mutants of Arabidopsis. Plant Physiol. 2001, 126:536-548.

11. Rosso MG, Li Y, Strizhov N, Reiss B, Dekker K, Weisshaar B. An Arabidopsis thaliana T-DNA mutagenized population (GABI-Kat) for flanking sequence tag-based reverse genetics. Plant Mol Biol 2003; 53: 247–259.

12. Schlaeppi K, Bodenhausen N, Buchala A, Mauch F, Reymond P: The glutathione-deficient mutant pad2-1 accumulates lower amounts of glucosinolates and is more susceptible to the insect herbivore *Spodoptera littoralis*. Plant J. 2008, 55:774-786.

13. Schuhegger R, Nafisi M, Mansourova M, Petersen BL, Olsen CE, Svatos A, Halkier BA, Glawischnig E: CYP71B15 (PAD3) catalyzes the final step in camalexin biosynthesis - Correction. Plant Physiol. 2007, 145:1086-1086.

14. Schulz E, Tohge T, Zuther E, Fernie AR, Hincha DK: Flavonoids are determinants of freezing tolerance and cold acclimation in *Arabidopsis thaliana*. Sci. Rep. 2016, 6.

15. Shirley B.W., Kubasek W.L., Storz G., Bruggemann E., Koornneef M., Ausubel FM., … Goodman H.M. Analysis of Arabidopsis mutants deficient in flavonoid biosynthesis.Plant J. 1995; 8, 659–71.

16. Stein M., Dittgen J., Sánchez-Rodríguez C., Hou B.H., Molina A., Schulze-Lefert P., … Somerville S. Arabidopsis PEN3/PDR8, an ATP binding cassette transporter, contributes to nonhost resistance to inappropriate pathogens that enter by direct penetration. Plant Cell. 2006; 18, 731–46.

17. Yamamoto S, Katagiri M, Maeno H, & Hayaishi O. Salicylate hydroxylase, a monooxygenase requiring flavin adenine. J. Biol. Chem. 1965; 240:3414–7.

18. Zhao YD, Hull AK, Gupta NR, Goss KA, Alonso J, Ecker JR, Normanly J, Chory J, Celenza JL: Trp-dependent auxin biosynthesis in Arabidopsis: involvement of cytochrome P450s CYP79B2 and CYP79B3. Genes & Dev. 2002; 16:3100-3112.

Table S2: Mean, median and range of reads per genotype groups for fungal and bacterial libraries.

	mean	median	min	max
Bacteria	6365	3277	7	44934
Fungi	6187	1762	26	57210

Table S3: Differences in alpha diversity in secondary metabolite and signaling mutants and their control. Analysis was performed using the Kruskal-Wallis test.

**Dataset/factor**	**Bacteria**		**Fungi**	
	observed	Shannon	observed	Shannon
**GLS**				
Col_0; *cyp79B3*	0.04*	n.s	--	--
Col_0; *cyp79B2cyp79B3*	n.s	n.s	0.025*	n.s
Col_0; *myb51*	n.s	n.s	0.01*	n.s
Col_0; *gsm1_1*	0.02*	n.s	n.s	n.s
Col_0; *tu3*	n.s	n.s	n.s	n.s
Col_0; *pad2_1*	0.04*	0.04*	--	--
Col_0; *Pen3_1NahG*	n.s	n.s	n.s	n.s
**FLVs**				
Ler_0; *tt7_7*	n.s	n.s	n.s	n.s
Ler_0; *tt3*	n.s	n.s	0.03*	
Ler_0; *tt5*	n.s	n.s	0.049*	
Ler_0; *tt3_1tt5_1*	n.s	n.s	0.049*	
Col_0; *pap1_D*	n.s	n.s	n.s	n.s
**DSMs**				
Col_0; *dde2*	0.04*	0.04*	n.s	n.s

Ler_0; *aba3_2*	n.s	n.s	n.s	0.03*
Col_0; *etr1_3*	n.s	n.s	n.s	n.s
Col_0; *35S_ERF*	n.s	n.s	n.s	n.s
Col_0; *fad3_2*	--	--	0.03*	n.s
Col_0; *fad7_1fad8_1*	n.s	n.s	n.s	n.s

Significance of test indicated as *** for p<0.001, ** p <0.01, *p<0.05

Table S4: Multivariate analysis of the deviance of generalized linear models fitting bacterial otus to secondary metabolite and signaling mutants genotypes.

Dataset	Factor		Res.Df	Df.diff	Dev	Dev<br>Pr(>Dev)
GLSmut	Intercept<br>Genotype		33<br>26	 7	 19637	 0.001 ***
FLV	Intercept<br>Genotype		18<br>14	 4	 10966	 0.001 ***
DSM	Intercept<br>Genotype		20<br>16	 4	 11596	 0.001 ***

Signif. codes: 0 '***' 0.001 '**' 0.01 '*' 0.05

Table S5: Multivariate analysis of the deviance of generalized linear models fitting fungal otus to secondary metabolite and signaling mutants and control genotypes.

Dataset	Factor	Res.Df	Df.diff	Dev	Dev<br>Pr(>Dev)
GLS	Intercept<br>Genotype	24<br>19	 5	 1997	 0.001 ***
FLV	Intercept<br>Genotype	17<br>13	 4	 1534	 0.001 ***
DSM	Intercept<br>Genotype	23<br>18	 5	 1950	 0.001 ***

Signif. codes: 0 '***' 0.001 '**' 0.01 '*' 0.05

Table S6: Proportional abundance (in percentage) of differentially affected genera in microbiome

Dataset		Bacteria	Fungi
**GLSs**	Enriched	7.39	12.98
	Depleted	4.63	3.89
	Total	12.02	16.87
**FLVs**	Enriched	2.74	10.09
	Depleted	1.49	13.53
	Total	4.23	23.62
**DSMs**	Enriched	11.04	27.85
	Depleted	9.21	3.52
	Total	20.25	31.37

Table S7: Differentially abundant root bacterial OTUs of Col_0 and GLS mutants as determined using DESeq2.

otu	FoldChange	Class	Family	Genus	Species	WT_Mutant	*Mutant*	Taxonomy_OTU
OTU_103	-4.542349509	Bacteroidia	Crocinitomicaceae	Fluviicola	NA	Col_0_cyp79B3	*cyp79B3*	Bacteroidia;Fluviicola;OTU_103
OTU_103	-5.420224198	Bacteroidia	Crocinitomicaceae	Fluviicola	NA	Col_0_cyp79B2cyp79B3	*cyp79B2cyp79B3*	Bacteroidia;Fluviicola;OTU_103
OTU_103	-4.937442654	Bacteroidia	Crocinitomicaceae	Fluviicola	NA	Col_0_myb51	*myb51*	Bacteroidia;Fluviicola;OTU_103
OTU_103	-6.035289414	Bacteroidia	Crocinitomicaceae	Fluviicola	NA	Col_0_gsm1_1	*gsm1_1*	Bacteroidia;Fluviicola;OTU_103
OTU_103	-4.500402596	Bacteroidia	Crocinitomicaceae	Fluviicola	NA	Col_0_TU3	*TU3*	Bacteroidia;Fluviicola;OTU_103
OTU_103	-5.371838741	Bacteroidia	Crocinitomicaceae	Fluviicola	NA	Col_0_pad2_1	*pad2_1*	Bacteroidia;Fluviicola;OTU_103
OTU_116	2.467139904	Subgroup 6	NA	NA	NA	Col_0_TU3	*TU3*	Subgroup 6;NA;OTU_116
OTU_1249	3.431695936	Acidimicrobiia	Ilumatobacteraceae	CL500-29 marine group	NA	Col_0_myb51	*myb51*	Acidimicrobiia;CL500-29 marine group;OTU_1249
OTU_13	-1.425399429	Actinobacteria	Nocardioidaceae	Nocardioides	Ambiguous_taxa	Col_0_cyp79B3	*cyp79B3*	Actinobacteria;Nocardioides;OTU_13
OTU_13	-1.950023779	Actinobacteria	Nocardioidaceae	Nocardioides	Ambiguous_taxa	Col_0_TU3	*TU3*	Actinobacteria;Nocardioides;OTU_13
OTU_13	-3.506744447	Actinobacteria	Nocardioidaceae	Nocardioides	uncultured bacterium	Col_0_cyp79B3	*cyp79B3*	Actinobacteria;Nocardioides;OTU_13
OTU_13	-4.041682424	Actinobacteria	Nocardioidaceae	Nocardioides	uncultured bacterium	Col_0_TU3	*TU3*	Actinobacteria;Nocardioides;OTU_13
OTU_140	-1.931579563	Thermoleophilia	uncultured	NA	NA	Col_0_TU3	*TU3*	Thermoleophilia;NA;OTU_140
OTU_149	-2.683288697	Actinobacteria	Frankiaceae	Jatrophihabitans	Ambiguous_taxa	Col_0_TU3	*TU3*	Actinobacteria;Jatrophihabitans;OTU_149

OTU_16	-1.253806548	Actinobacteria	Streptomycetaceae	Streptomyces	NA	Col_0_cyp79B3	*cyp79B3*	Actinobacteria;Streptomyces;OTU_16
OTU_16	-1.7159077	Actinobacteria	Streptomycetaceae	Streptomyces	NA	Col_0_cyp79B2cyp79B3	*cyp79B2cyp79B3*	Actinobacteria;Streptomyces;OTU_16
OTU_16	-1.561806197	Actinobacteria	Streptomycetaceae	Streptomyces	NA	Col_0_pad2_1	*pad2_1*	Actinobacteria;Streptomyces;OTU_16
OTU_17	1.840243359	Alphaproteobacteria	Rhizobiaceae	ANP_Rhizobium	NA	Col_0_cyp79B3	*cyp79B3*	Alphaproteobacteria;ANP_Rhizobium;OTU_17
OTU_171	1.767400207	Planctomycetacia	Gemmataceae	Gemmata	uncultured bacterium	Col_0_cyp79B3	*cyp79B3*	Planctomycetacia;Gemmata;OTU_171
OTU_178	-2.303724324	Actinobacteria	Sporichthyaceae	NA	uncultured bacterium	Col_0_TU3	*TU3*	Actinobacteria;NA;OTU_178
OTU_18	1.541634276	Phycisphaerae	WD2101 soil group	NA	uncultured bacterium	Col_0_cyp79B3	*cyp79B3*	Phycisphaerae;NA;OTU_18
OTU_192	-1.810123746	Thermoleophilia	uncultured	NA	uncultured bacterium	Col_0_cyp79B3	*cyp79B3*	Thermoleophilia;NA;OTU_192
OTU_20	1.199038787	Verrucomicrobiae	Chthoniobacteraceae	Cand. Udaeobacter	Ambiguous_taxa	Col_0_myb51	*myb51*	Verrucomicrobiae;Cand. Udaeobacter;OTU_20
OTU_201	7.822042756	Bacteroidia	Crocinitomicaceae	Fluviicola	uncultured bacterium	Col_0_cyp79B3	*cyp79B3*	Bacteroidia;Fluviicola;OTU_201
OTU_202	1.472077138	Verrucomicrobiae	Chthoniobacteraceae	Cand. Udaeobacter	Ambiguous_taxa	Col_0_TU3	*TU3*	Verrucomicrobiae;Cand. Udaeobacter;OTU_202
OTU_230	-2.252969729	Gammaproteobacteria	Rhodanobacteraceae	Rhodanobacter	NA	Col_0_Pen3_1NahG	*Pen3_1NahG*	Gammaproteobacteria;Rhodanobacter;OTU_230
OTU_248	3.524395175	Bacteroidia	Chitinophagaceae	Niastella	uncultured bacterium	Col_0_gsm1_1	*gsm1_1*	Bacteroidia;Niastella;OTU_248
OTU_28	-1.919346745	Actinobacteria	Intrasporangiaceae	Terrabacter	Ambiguous_taxa	Col_0_TU3	*TU3*	Actinobacteria;Terrabacter;OTU_28
OTU_28	-1.177575235	Actinobacteria	Intrasporangiaceae	Terrabacter	Ambiguous_taxa	Col_0_Pen3_1NahG	*Pen3_1NahG*	Actinobacteria;Terrabacter;OTU_28
OTU_283	1.827177161	Verrucomicrobiae	Xiphinematobacteraceae	Cand. Xiphinematobacter	uncultured bacterium	Col_0_cyp79B2cyp79B3	*cyp79B2cyp79B3*	Verrucomicrobiae;Cand. Xiphinematobacter;OTU_283
OTU_283	2.10160609	Verrucomicrobiae	Xiphinematobacteraceae	Cand. Xiphinematobacter	uncultured bacterium	Col_0_myb51	*myb51*	Verrucomicrobiae;Cand. Xiphinematobacter;OTU_283
OTU_296	3.072930199	Verrucomicrobiae	Verrucomicrobiaceae	Roseimicrobium	NA	Col_0_cyp79B2cyp79B3	*cyp79B2cyp79B3*	Verrucomicrobiae;Roseimicrobium;OTU_296
OTU_30	-1.472208325	Bacteroidia	Chitinophagaceae	NA	uncultured bacterium	Col_0_Pen3_1NahG	*Pen3_1NahG*	Bacteroidia;NA;OTU_30
OTU_302	2.405990292	Planctomycetacia	Isosphaeraceae	Singulisphaera	Ambiguous_taxa	Col_0_cyp79B3	*cyp79B3*	Planctomycetacia;Singulisphaera;OTU_302
OTU_318	-3.107578425	Gammaproteobacteria	Burkholderiaceae	Massilia	uncultured bacterium	Col_0_cyp79B3	*cyp79B3*	Gammaproteobacteria;Massilia;OTU_318
OTU_318	-2.81418065	Gammaproteobacteria	Burkholderiaceae	Massilia	uncultured bacterium	Col_0_TU3	*TU3*	Gammaproteobacteria;Massilia;OTU_318
OTU_331	4.004059832	Gammaproteobacteria	Pseudomonadaceae	Pseudomonas	Ambiguous_taxa	Col_0_cyp79B2cyp79B3	*cyp79B2cyp79B3*	Gammaproteobacteria;Pseudomonas;OTU_331
OTU_331	2.871631158	Gammaproteobacteria	Pseudomonadaceae	Pseudomonas	Ambiguous_taxa	Col_0_pad2_1	*pad2_1*	Gammaproteobacteria;Pseudomonas;OTU_331
OTU_331	3.078411729	Gammaproteobacteria	Pseudomonadaceae	Pseudomonas	Ambiguous_taxa	Col_0_Pen3_1NahG	*Pen3_1NahG*	Gammaproteobacteria;Pseudomonas;OTU_331
OTU_339	1.429228846	Planctomycetacia	uncultured	NA	Ambiguous_taxa	Col_0_cyp79B3	*cyp79B3*	Planctomycetacia;NA;OTU_339
OTU_339	2.206495622	Planctomycetacia	uncultured	Ambiguous_taxa	Ambiguous_taxa	Col_0_cyp79B2cyp79B3	*cyp79B2cyp79B3*	Planctomycetacia;Ambiguous_taxa;OTU_339
OTU_339	1.722063162	Planctomycetacia	uncultured	Ambiguous_taxa	Ambiguous_taxa	Col_0_TU3	*TU3*	Planctomycetacia;Ambiguous_taxa;OTU_339
OTU_341	2.583272644	Planctomycetacia	Pirellulaceae	Pirellula	uncultured bacterium	Col_0_gsm1_1	*gsm1_1*	Planctomycetacia;Pirellula;OTU_341
OTU_3486	-2.27302632	Actinobacteria	Intrasporangiaceae	Lapillicoccus	NA	Col_0_cyp79B3	*cyp79B3*	Actinobacteria;Lapillicoccus;OTU_3486

OTU_3486	-1.776400889	Actinobacteria	Intrasporangiaceae	Lapillicoccus	NA	Col_0_cyp79B2cyp79B3	*cyp79B2cyp79B3*	Actinobacteria;Lapillicoccus;OTU_3486
OTU_3486	-1.86520495	Actinobacteria	Intrasporangiaceae	Lapillicoccus	NA	Col_0_pad2_1	*pad2_1*	Actinobacteria;Lapillicoccus;OTU_3486
OTU_3486	-1.644846185	Actinobacteria	Intrasporangiaceae	Lapillicoccus	NA	Col_0_myb51	*myb51*	Actinobacteria;Lapillicoccus;OTU_3486
OTU_3486	-2.048467064	Actinobacteria	Intrasporangiaceae	Lapillicoccus	NA	Col_0_TU3	*TU3*	Actinobacteria;Lapillicoccus;OTU_3486
OTU_38	1.515570347	Verrucomicrobiae	Chthoniobacteraceae	Cand. Udaeobacter	NA	Col_0_TU3	*TU3*	Verrucomicrobiae;Cand. Udaeobacter;OTU_38
OTU_38	1.595455165	Verrucomicrobiae	Chthoniobacteraceae	Cand. Udaeobacter	uncultured bacterium	Col_0_TU3	*TU3*	Verrucomicrobiae;Cand. Udaeobacter;OTU_38
OTU_393	-4.476236285	Chloroflexia	JG30-KF-CM45	NA	uncultured bacterium	Col_0_TU3	*TU3*	Chloroflexia;NA;OTU_393
OTU_405	-1.900991809	Thermoleophilia	67-14	NA	uncultured bacterium	Col_0_cyp79B3	*cyp79B3*	Thermoleophilia;NA;OTU_405
OTU_405	-1.774101507	Thermoleophilia	67-14	NA	uncultured bacterium	Col_0_TU3	*TU3*	Thermoleophilia;NA;OTU_405
OTU_416	-2.600997912	Bacilli	Paenibacillaceae	Ammoniphilus	uncultured bacterium	Col_0_TU3	*TU3*	Bacilli;Ammoniphilus;OTU_416
OTU_45	-1.901418064	Actinobacteria	Nocardioidaceae	Nocardioides	NA	Col_0_cyp79B3	*cyp79B3*	Actinobacteria;Nocardioides;OTU_45
OTU_45	-1.821604154	Actinobacteria	Nocardioidaceae	Nocardioides	NA	Col_0_cyp79B2cyp79B3	*cyp79B2cyp79B3*	Actinobacteria;Nocardioides;OTU_45
OTU_45	-1.501287725	Actinobacteria	Nocardioidaceae	Nocardioides	NA	Col_0_myb51	*myb51*	Actinobacteria;Nocardioides;OTU_45
OTU_45	-1.219918718	Actinobacteria	Nocardioidaceae	Nocardioides	NA	Col_0_Pen3_1NahG	*Pen3_1NahG*	Actinobacteria;Nocardioides;OTU_45
OTU_47	2.692821615	Deltaproteobacteria	Myxococcaceae	Corallococcus	Ambiguous_taxa	Col_0_pad2_1	*pad2_1*	Deltaproteobacteria;Corallococcus;OTU_47
OTU_502	2.978902216	Planctomycetacia	uncultured	NA	uncultured bacterium	Col_0_cyp79B2cyp79B3	*cyp79B2cyp79B3*	Planctomycetacia;NA;OTU_502
OTU_502	3.086945112	Planctomycetacia	uncultured	NA	uncultured bacterium	Col_0_pad2_1	*pad2_1*	Planctomycetacia;NA;OTU_502
OTU_517	-6.556175447	Gammaproteobacteria	Burkholderiaceae	Herminiimonas	uncultured bacterium	Col_0_cyp79B3	*cyp79B3*	Gammaproteobacteria;Herminiimonas;OTU_517
OTU_517	-7.851766957	Gammaproteobacteria	Burkholderiaceae	Herminiimonas	uncultured bacterium	Col_0_Pen3_1NahG	*Pen3_1NahG*	Gammaproteobacteria;Herminiimonas;OTU_517
OTU_519	-5.141803869	Gammaproteobacteria	Burkholderiaceae	Noviherbaspirillum	uncultured bacterium	Col_0_Pen3_1NahG	*Pen3_1NahG*	Gammaproteobacteria;Noviherbaspirillum;OTU_519
OTU_5218	2.873316068	Planctomycetacia	uncultured	NA	uncultured Planctomyces sp.	Col_0_cyp79B3	*cyp79B3*	Planctomycetacia;NA ;OTU_5218
OTU_533	-1.996227414	Thermoleophilia	67-14	NA	uncultured bacterium	Col_0_cyp79B3	*cyp79B3*	Thermoleophilia;NA;OTU_533
OTU_54	-7.987988827	Alphaproteobacteria	Azospirillaceae	Azospirillum	NA	Col_0_cyp79B3	*cyp79B3*	Alphaproteobacteria;Azospirillum;OTU_54
OTU_54	-8.359625792	Alphaproteobacteria	Azospirillaceae	Azospirillum	NA	Col_0_cyp79B2cyp79B3	*cyp79B2cyp79B3*	Alphaproteobacteria;Azospirillum;OTU_54
OTU_54	-5.513446439	Alphaproteobacteria	Azospirillaceae	Azospirillum	NA	Col_0_myb51	*myb51*	Alphaproteobacteria;Azospirillum;OTU_54
OTU_54	-8.974295342	Alphaproteobacteria	Azospirillaceae	Azospirillum	NA	Col_0_gsm1_1	*gsm1_1*	Alphaproteobacteria;Azospirillum;OTU_54
OTU_54	-7.369812203	Alphaproteobacteria	Azospirillaceae	Azospirillum	NA	Col_0_TU3	*TU3*	Alphaproteobacteria;Azospirillum;OTU_54
OTU_54	-8.31207967	Alphaproteobacteria	Azospirillaceae	Azospirillum	NA	Col_0_pad2_1	*pad2_1*	Alphaproteobacteria;Azospirillum;OTU_54
OTU_54	-5.566068435	Alphaproteobacteria	Azospirillaceae	Azospirillum	NA	Col_0_Pen3_1NahG	*Pen3_1NahG*	Alphaproteobacteria;Azospirillum;OTU_54
OTU_56	-1.706526994	Actinobacteria	Nocardioidaceae	Nocardioides	Ambiguous_taxa	Col_0_cyp79B3	*cyp79B3*	Actinobacteria;Nocardioides;OTU_56
OTU_56	-1.624386021	Actinobacteria	Nocardioidaceae	Nocardioides	Ambiguous_taxa	Col_0_myb51	*myb51*	Actinobacteria;Nocardioides;OTU_56

OTU_56	-2.087546657	Actinobacteria	Nocardioidaceae	Nocardioides	Ambiguous_taxa	Col_0_TU3	*TU3*	Actinobacteria;Nocardioides;OTU_56
OTU_56	-1.146681845	Actinobacteria	Nocardioidaceae	Nocardioides	Ambiguous_taxa	Col_0_Pen3_1NahG	*Pen3_1NahG*	Actinobacteria;Nocardioides;OTU_56
OTU_5687	5.521524661	Verrucomicrobiae	Pedosphaeraceae	NA	uncultured bacterium	Col_0_cyp79B3	*cyp79B3*	Verrucomicrobiae;NA;OTU_5687
OTU_576	3.90965751	Phycisphaerae	WD2101 soil group	NA	uncultured bacterium	Col_0_cyp79B3	*cyp79B3*	Phycisphaerae;NA;OTU_576
OTU_58	-1.768125938	Thermoleophilia	67-14	NA	uncultured bacterium	Col_0_cyp79B3	*cyp79B3*	Thermoleophilia;NA;OTU_58
OTU_58	-1.834200722	Thermoleophilia	67-14	NA	uncultured bacterium	Col_0_cyp79B2cyp79B3	*cyp79B2cyp79B3*	Thermoleophilia;NA;OTU_58
OTU_58	-1.392777792	Thermoleophilia	67-14	NA	uncultured bacterium	Col_0_gsm1_1	*gsm1_1*	Thermoleophilia;NA;OTU_58
OTU_58	-1.490507411	Thermoleophilia	67-14	NA	uncultured bacterium	Col_0_TU3	*TU3*	Thermoleophilia;NA;OTU_58
OTU_58	-2.091850896	Thermoleophilia	67-14	NA	uncultured bacterium	Col_0_pad2_1	*pad2_1*	Thermoleophilia;NA;OTU_58
OTU_6081	6.011093803	Bacteroidia	Flavobacteriaceae	Flavobacterium	uncultured bacterium	Col_0_myb51	*myb51*	Bacteroidia;Flavobacterium;OTU_6081
OTU_6428	5.909241049	Gammaproteobacteria	Burkholderiaceae	Variovorax	NA	Col_0_cyp79B2cyp79B3	*cyp79B2cyp79B3*	Gammaproteobacteria;Variovorax;OTU_6428
OTU_6428	6.159833718	Gammaproteobacteria	Burkholderiaceae	Variovorax	NA	Col_0_pad2_1	*pad2_1*	Gammaproteobacteria;Variovorax;OTU_6428
OTU_667	-4.772592708	Bacteroidia	Flavobacteriaceae	Flavobacterium	NA	Col_0_cyp79B3	*cyp79B3*	Bacteroidia;Flavobacterium;OTU_667
OTU_667	-5.566479238	Bacteroidia	Flavobacteriaceae	Flavobacterium	NA	Col_0_TU3	*TU3*	Bacteroidia;Flavobacterium;OTU_667
OTU_667	-4.410053853	Bacteroidia	Flavobacteriaceae	Flavobacterium	NA	Col_0_Pen3_1NahG	*Pen3_1NahG*	Bacteroidia;Flavobacterium;OTU_667
OTU_68	-1.800104448	Thermoleophilia	uncultured	NA	uncultured bacterium	Col_0_TU3	*TU3*	Thermoleophilia;NA;OTU_68
OTU_70	-2.161259645	Actinobacteria	Nocardioidaceae	Nocardioides	uncultured bacterium	Col_0_TU3	*TU3*	Actinobacteria;Nocardioides;OTU_70
OTU_72	-1.99864107	Actinobacteria	Geodermatophilaceae	Blastococcus	uncultured bacterium	Col_0_TU3	*TU3*	Actinobacteria;Blastococcus;OTU_72
OTU_72	-2.623025461	Actinobacteria	Geodermatophilaceae	Blastococcus	uncultured bacterium	Col_0_pad2_1	*pad2_1*	Actinobacteria;Blastococcus;OTU_72
OTU_7238	-4.457283601	Verrucomicrobiae	Verrucomicrobiaceae	Prosthecobacter	uncultured bacterium	Col_0_cyp79B3	*cyp79B3*	Verrucomicrobiae;Prosthecobacter;OTU_7238
OTU_7238	-5.133967759	Verrucomicrobiae	Verrucomicrobiaceae	Prosthecobacter	uncultured bacterium	Col_0_Pen3_1NahG	*Pen3_1NahG*	Verrucomicrobiae;Prosthecobacter;OTU_7238
OTU_769	4.376185227	Planctomycetacia	Gemmataceae	NA	uncultured bacterium	Col_0_cyp79B3	*cyp79B3*	Planctomycetacia;NA;OTU_769
OTU_81	2.049799938	Verrucomicrobiae	Chthoniobacteraceae	Cand. Udaeobacter	uncultured Verrucomicrobia bacterium	Col_0_cyp79B3	*cyp79B3*	Verrucomicrobiae;Cand. Udaeobacter;OTU_81
OTU_82	-1.942821908	Actinobacteria	Nocardioidaceae	Marmoricola	Ambiguous_taxa	Col_0_cyp79B3	*cyp79B3*	Actinobacteria;Marmoricola;OTU_82
OTU_874	-4.719531858	Gammaproteobacteria	Xanthomonadaceae	Stenotrophomonas	Stenotrophomonas maltophilia	Col_0_TU3	*TU3*	Gammaproteobacteria;Stenotrophomonas;OTU_874
OTU_94	3.609115262	Alphaproteobacteria	Sphingomonadaceae	Sphingopyxis	NA	Col_0_cyp79B3	*cyp79B3*	Alphaproteobacteria;Sphingopyxis;OTU_94
OTU_94	4.742379887	Alphaproteobacteria	Sphingomonadaceae	Sphingopyxis	NA	Col_0_cyp79B2cyp79B3	*cyp79B2cyp79B3*	Alphaproteobacteria;Sphingopyxis;OTU_94
OTU_97	-2.29673468	Actinobacteria	Nocardioidaceae	Nocardioides	uncultured bacterium	Col_0_cyp79B3	*cyp79B3*	Actinobacteria;Nocardioides;OTU_97
OTU_97	-2.459144791	Actinobacteria	Nocardioidaceae	Nocardioides	uncultured bacterium	Col_0_TU3	*TU3*	Actinobacteria;Nocardioides;OTU_97
OTU_97	-1.558001195	Actinobacteria	Nocardioidaceae	Nocardioides	uncultured bacterium	Col_0_Pen3_1NahG	*Pen3_1NahG*	Actinobacteria;Nocardioides;OTU_97

Table S8: Differentially abundant root bacterial OTUs of Col_0 and Ler_0 and FLV mutants as determined using DESeq2.

otu	FoldChange	Class	Family	Genus	Species	WT_Mutant	Mutant	Taxonomy_OTU
OTU_38	-2.97858663	Verrucomicrobiae	Chthoniobacteraceae	Cand. Udaeobacter	NA	Ler_0_tt7_7	*tt7_7*	Verrucomicrobiae;Cand. Udaeobacter;OTU_38
OTU_3	1.743267866	Actinobacteria	Micrococcaceae	Pseudarthrobacter	NA	Ler_0_tt7_7	*tt7_7*	Actinobacteria;Pseudarthrobacter;OTU_3
OTU_126	-3.14119781	Blastocatellia (Subgroup 4)	Blastocatellaceae	JGI 0001001-H03	NA	Ler_0_tt7_7	*tt7_7*	Blastocatellia (Subgrp4);JGI 0001001-H03;OTU_126
OTU_667	-6.93478856	Bacteroidia	Flavobacteriaceae	Flavobacterium	NA	Ler_0_tt7_7	*tt7_7*	Bacteroidia;Flavobacterium;OTU_667
OTU_43	1.962719485	Gammaproteobacteria	Burkholderiaceae	Massilia	uncultured Telluria sp.	Ler_0_tt7_7	*tt7_7*	Gammaproteobacteria;Massilia;OTU_43
OTU_117	-7.61194832	Gammaproteobacteria	Rhodanobacteraceae	Rhodanobacter	Ambiguous_taxa	Ler_0_tt7_7	*tt7_7*	Gammaproteobacteria;Rhodanobacter;OTU_117
OTU_6671	-7.28576301	Bacteroidia	Flavobacteriaceae	Flavobacterium	NA	Ler_0_tt5	*tt5*	Bacteroidia;Flavobacterium;OTU_6671
OTU_7238	-4.98047905	Verrucomicrobiae	Verrucomicrobiaceae	Prosthecobacter	uncultured bacterium	Col_0_pap1_D	*pap1_D*	Verrucomicrobiae;Prosthecobacter;OTU_7238
OTU_28	-2.07020319	Actinobacteria	Intrasporangiaceae	Terrabacter	Ambiguous_taxa	Col_0_pap1_D	*pap1_D*	Actinobacteria;Terrabacter;OTU_28
OTU_103	-4.73322446	Bacteroidia	Crocinitomicaceae	Fluviicola	NA	Col_0_pap1_D	*pap1_D*	Bacteroidia;Fluviicola;OTU_103
OTU_6672	-5.35514599	Bacteroidia	Flavobacteriaceae	Flavobacterium	NA	Col_0_pap1_D	*pap1_D*	Bacteroidia;Flavobacterium;OTU_6672
OTU_519	-4.32498315	Gammaproteobacteria	Burkholderiaceae	Noviherbaspirillum	uncultured bacterium	Col_0_pap1_D	*pap1_D*	Gammaproteobacteria;Noviherbaspirillum;OTU_519
OTU_318	-2.82480782	Gammaproteobacteria	Burkholderiaceae	Massilia	uncultured bacterium	Col_0_pap1_D	*pap1_D*	Gammaproteobacteria;Massilia;OTU_318
OTU_54	-8.6981714	Alphaproteobacteria	Azospirillaceae	Azospirillum	NA	Col_0_pap1_D	*pap1_D*	Alphaproteobacteria;Azospirillum;OTU_54

Table S9: Differentially abundant root bacterial OTUs of Col_0 and Ler_0 and DSMs mutants as determined using DESeq2.

otu	FoldChange	Class	Family	Genus	Species	WT_Mutant	Mutant	Taxonomy_OTU
OTU_103	-3.476457382	Bacteroidia	Crocinitomicaceae	Fluviicola	NA	Col_0_dde2	*dde2*	Bacteroidia;Fluviicola;OTU_103
OTU_103	-5.581118515	Bacteroidia	Crocinitomicaceae	Fluviicola	NA	Col_0_etr1_3	*etr1_3*	Bacteroidia;Fluviicola;OTU_103
OTU_103	-5.150983934	Bacteroidia	Crocinitomicaceae	Fluviicola	NA	Col_0_35S_ERF	*35S_ERF*	Bacteroidia;Fluviicola;OTU_103
OTU_109	6.001387306	Oxyphotobacteria	Leptolyngbyaceae	Leptolyngbya Es-Yyy1000	uncultured bacterium	Col_0_dde2	*dde2*	Oxyphotobacteria;Leptolyngbya Es-Yyy1000;OTU_109
OTU_117	2.471001666	Gammaproteobacteria	Rhodanobacteraceae	Rhodanobacter	Ambiguous_taxa	Col_0_dde2	*dde2*	Gammaproteobacteria;Rhodanobacter;OTU_117
OTU_119	-1.680414929	Actinobacteria	Mycobacteriaceae	Mycobacterium	NA	Col_0_etr1_3	*etr1_3*	Actinobacteria;Mycobacterium;OTU_119
OTU_13	-1.648999374	Actinobacteria	Nocardioidaceae	Nocardioides	Ambiguous_taxa	Col_0_etr1_3	*etr1_3*	Actinobacteria;Nocardioides;OTU_13
OTU_1325	-2.515268687	Actinobacteria	Nocardioidaceae	Nocardioides	uncultured bacterium	Col_0_fad7_1fad8_1	*fad7_1fad8_1*	Actinobacteria;Nocardioides;OTU_1325
OTU_136	10.18444255	Gammaproteobacteria	Cellvibrionaceae	Cellvibrio	uncultured bacterium	Col_0_dde2	*dde2*	Gammaproteobacteria;Cellvibrio;OTU_136

OTU_136	6.54864941	Gammaproteobacteria	Cellvibrionaceae	Cellvibrio	uncultured bacterium	Col_0_fad7_1fad8_1	*fad7_1fad8_1*	Gammaproteobacteria;Cellvibrio;OTU_136
OTU_140	-1.170258694	Thermoleophilia	uncultured	NA	NA	Col_0_fad7_1fad8_1	*fad7_1fad8_1*	Thermoleophilia;NA;OTU_140
OTU_142	4.251773128	Oxyphotobacteria	Nostocaceae	Cylindrospermum PCC-7417	NA	Col_0_dde2	*dde2*	Oxyphotobacteria;Cylindrospermum PCC-7417;OTU_142
OTU_149	-2.071439831	Actinobacteria	Frankiaceae	Jatrophihabitans	Ambiguous_taxa	Col_0_etr1_3	*etr1_3*	Actinobacteria;Jatrophihabitans;OTU_149
OTU_16	-1.742746923	Actinobacteria	Streptomycetaceae	Streptomyces	NA	Col_0_etr1_3	*etr1_3*	Actinobacteria;Streptomyces;OTU_16
OTU_166	1.582562117	Gammaproteobacteria	SC-I-84	NA	uncultured bacterium	Col_0_fad7_1fad8_1	*fad7_1fad8_1*	Gammaproteobacteria;NA;OTU_166
OTU_17	3.137022059	Alphaproteobacteria	Rhizobiaceae	ANP-Rhizobium	NA	Col_0_dde2	*dde2*	Alphaproteobacteria;ANP-Rhizobium;OTU_17
OTU_17	1.380216626	Alphaproteobacteria	Rhizobiaceae	ANP-Rhizobium	NA	Col_0_fad7_1fad8_1	*fad7_1fad8_1*	Alphaproteobacteria;ANP-Rhizobium;OTU_17
OTU_173	2.882582102	Alphaproteobacteria	Caulobacteraceae	Phenylobacterium	Ambiguous_taxa	Col_0_dde2	*dde2*	Alphaproteobacteria;Phenylobacterium;OTU_173
OTU_1779	4.504999603	Planctomycetacia	Gemmataceae	Gemmata	uncultured bacterium	Col_0_etr1_3	*etr1_3*	Planctomycetacia;Gemmata;OTU_1779
OTU_178	-1.970998019	Actinobacteria	Sporichthyaceae	NA	uncultured bacterium	Col_0_etr1_3	*etr1_3*	Actinobacteria;NA;OTU_178
OTU_178	-1.509206617	Actinobacteria	Sporichthyaceae	NA	uncultured bacterium	Col_0_fad7_1fad8_1	*fad7_1fad8_1*	Actinobacteria;NA;OTU_178
OTU_190	-2.56966577	Verrucomicrobiae	Chthoniobacteraceae	Cand. Udaeobacter	NA	Ler_0_aba3_2	*aba3_2*	Verrucomicrobiae;Cand. Udaeobacter;OTU_190
OTU_20	-1.492533387	Verrucomicrobiae	Chthoniobacteraceae	Cand. Udaeobacter	Ambiguous_taxa	Ler_0_aba3_2	*aba3_2*	Verrucomicrobiae;Cand. Udaeobacter;OTU_20
OTU_201	10.49881922	Bacteroidia	Crocinitomicaceae	Fluviicola	uncultured bacterium	Col_0_dde2	*dde2*	Bacteroidia;Fluviicola;OTU_201
OTU_201	8.476016863	Bacteroidia	Crocinitomicaceae	Fluviicola	uncultured bacterium	Col_0_fad7_1fad8_1	*fad7_1fad8_1*	Bacteroidia;Fluviicola;OTU_201
OTU_202	1.446767094	Verrucomicrobiae	Chthoniobacteraceae	Cand. Udaeobacter	Ambiguous_taxa	Col_0_etr1_3	*etr1_3*	Verrucomicrobiae;Cand. Udaeobacter;OTU_202
OTU_21	1.454990613	Alphaproteobacteria	Methyloligellaceae	NA	Ambiguous_taxa	Col_0_fad7_1fad8_1	*fad7_1fad8_1*	Alphaproteobacteria;NA;OTU_21
OTU_228	7.57543183	Bacteroidia	Sphingobacteriaceae	Pedobacter	NA	Col_0_dde2	*dde2*	Bacteroidia;Pedobacter;OTU_228
OTU_28	-1.535524249	Actinobacteria	Intrasporangiaceae	Terrabacter	Ambiguous_taxa	Col_0_etr1_3	*etr1_3*	Actinobacteria;Terrabacter;OTU_28
OTU_281	6.117288618	Alphaproteobacteria	Beijerinckiaceae	Bosea	NA	Col_0_dde2	*dde2*	Alphaproteobacteria;Bosea;OTU_281
OTU_3	1.425081056	Actinobacteria	Micrococcaceae	Pseudarthrobacter	NA	Ler_0_aba3_2	*aba3_2*	Actinobacteria;Pseudarthrobacter;OTU_3
OTU_3	0.832307889	Actinobacteria	Micrococcaceae	Pseudarthrobacter	NA	Col_0_fad7_1fad8_1	*fad7_1fad8_1*	Actinobacteria;Pseudarthrobacter;OTU_3
OTU_318	-2.848801354	Gammaproteobacteria	Burkholderiaceae	Massilia	uncultured bacterium	Col_0_fad7_1fad8_1	*fad7_1fad8_1*	Gammaproteobacteria;Massilia;OTU_318
OTU_331	4.755934045	Gammaproteobacteria	Pseudomonadaceae	Pseudomonas	Ambiguous_taxa	Col_0_dde2	*dde2*	Gammaproteobacteria;Pseudomonas;OTU_331
OTU_331	3.424214913	Gammaproteobacteria	Pseudomonadaceae	Pseudomonas	Ambiguous_taxa	Col_0_etr1_3	*etr1_3*	Gammaproteobacteria;Pseudomonas;OTU_331
OTU_331	2.496552952	Gammaproteobacteria	Pseudomonadaceae	Pseudomonas	Ambiguous_taxa	Col_0_35S_ERF	*35S_ERF*	Gammaproteobacteria;Pseudomonas;OTU_331
OTU_339	1.889641254	Planctomycetacia	uncultured	Ambiguous_taxa	Ambiguous_taxa	Col_0_etr1_3	*etr1_3*	Planctomycetacia;Ambiguous_taxa;OTU_339
OTU_341	2.45105702	Planctomycetacia	Pirellulaceae	Pirellula	uncultured bacterium	Col_0_etr1_3	*etr1_3*	Planctomycetacia;Pirellula;OTU_341
OTU_3486	-1.481275263	Actinobacteria	Intrasporangiaceae	Lapillicoccus	NA	Col_0_etr1_3	*etr1_3*	Actinobacteria;Lapillicoccus;OTU_3486
OTU_3486	-1.867128487	Actinobacteria	Intrasporangiaceae	Lapillicoccus	NA	Col_0_35S_ERF	*35S_ERF*	Actinobacteria;Lapillicoccus;OTU_3486

OTU_3620	2.640336749	Verrucomicrobiae	Chthoniobacteraceae	Cand. Udaeobacter	NA	Col_0_etr1_3	*etr1_3*	Verrucomicrobiae;Cand. Udaeobacter;OTU_3620
OTU_38	-1.921534299	Verrucomicrobiae	Chthoniobacteraceae	Cand. Udaeobacter	NA	Ler_0_aba3_2	*aba3_2*	Verrucomicrobiae;Cand. Udaeobacter;OTU_38
OTU_39	-2.345233006	Verrucomicrobiae	Chthoniobacteraceae	Cand. Udaeobacter	uncultured bacterium	Ler_0_aba3_2	*aba3_2*	Verrucomicrobiae;Cand. Udaeobacter;OTU_39
OTU_393	-5.025361933	Chloroflexia	JG30-KF-CM45	NA	uncultured bacterium	Col_0_etr1_3	*etr1_3*	Chloroflexia;NA;OTU_393
OTU_40	5.016834157	Alphaproteobacteria	Caulobacteraceae	Brevundimonas	Ambiguous_taxa	Col_0_dde2	*dde2*	Alphaproteobacteria;Brevundimonas;OTU_40
OTU_404	1.877875346	Gammaproteobacteria	Unknown Family	Acidibacter	NA	Col_0_35S_ERF	*35S_ERF*	Gammaproteobacteria;Acidibacter;OTU_404
OTU_405	-1.642863245	Thermoleophilia	67-14	NA	uncultured bacterium	Col_0_etr1_3	*etr1_3*	Thermoleophilia;NA;OTU_405
OTU_405	-1.342449962	Thermoleophilia	67-14	NA	uncultured bacterium	Col_0_fad7_1fad8_1	*fad7_1fad8_1*	Thermoleophilia;NA;OTU_405
OTU_43	2.133889257	Gammaproteobacteria	Burkholderiaceae	Massilia	uncultured Telluria sp.	Col_0_dde2	*dde2*	Gammaproteobacteria;Massilia;OTU_43
OTU_44	-1.973149687	Thermoleophilia	Gaiellaceae	Gaiella	NA	Col_0_etr1_3	*etr1_3*	Thermoleophilia;Gaiella;OTU_44
OTU_5	0.784100883	Alphaproteobacteria	Sphingomonadaceae	Sphingomonas	uncultured bacterium	Col_0_fad7_1fad8_1	*fad7_1fad8_1*	Alphaproteobacteria;Sphingomonas;OTU_5
OTU_517	-7.586986764	Gammaproteobacteria	Burkholderiaceae	Herminiimonas	uncultured bacterium	Col_0_dde2	*dde2*	Gammaproteobacteria;Herminiimonas;OTU_517
OTU_517	-7.587464301	Gammaproteobacteria	Burkholderiaceae	Herminiimonas	uncultured bacterium	Col_0_etr1_3	*etr1_3*	Gammaproteobacteria;Herminiimonas;OTU_517
OTU_517	-5.615771579	Gammaproteobacteria	Burkholderiaceae	Herminiimonas	uncultured bacterium	Col_0_35S_ERF	*35S_ERF*	Gammaproteobacteria;Herminiimonas;OTU_517
OTU_519	-6.648444506	Gammaproteobacteria	Burkholderiaceae	Noviherbaspirillum	uncultured bacterium	Ler_0_aba3_2	*aba3_2*	Gammaproteobacteria;Noviherbaspirillum;OTU_519
OTU_519	-3.199648685	Gammaproteobacteria	Burkholderiaceae	Noviherbaspirillum	uncultured bacterium	Col_0_fad7_1fad8_1	*fad7_1fad8_1*	Gammaproteobacteria;Noviherbaspirillum;OTU_519
OTU_533	-1.565100179	Thermoleophilia	67-14	NA	uncultured bacterium	Col_0_fad7_1fad8_1	*fad7_1fad8_1*	Thermoleophilia;NA;OTU_533
OTU_54	-6.893268926	Alphaproteobacteria	Azospirillaceae	Azospirillum	NA	Col_0_dde2	*dde2*	Alphaproteobacteria;Azospirillum;OTU_54
OTU_54	-8.531780225	Alphaproteobacteria	Azospirillaceae	Azospirillum	NA	Col_0_etr1_3	*etr1_3*	Alphaproteobacteria;Azospirillum;OTU_54
OTU_54	-8.451564064	Alphaproteobacteria	Azospirillaceae	Azospirillum	NA	Col_0_35S_ERF	*35S_ERF*	Alphaproteobacteria;Azospirillum;OTU_54
OTU_54	-9.380030979	Alphaproteobacteria	Azospirillaceae	Azospirillum	NA	Col_0_fad7_1fad8_1	*fad7_1fad8_1*	Alphaproteobacteria;Azospirillum;OTU_54
OTU_56	-2.034446047	Actinobacteria	Nocardioidaceae	Nocardioides	Ambiguous_taxa	Col_0_etr1_3	*etr1_3*	Actinobacteria;Nocardioides;OTU_56
OTU_56	-1.482947842	Actinobacteria	Nocardioidaceae	Nocardioides	Ambiguous_taxa	Col_0_fad7_1fad8_1	*fad7_1fad8_1*	Actinobacteria;Nocardioides;OTU_56
OTU_5687	5.680788198	Verrucomicrobiae	Pedosphaeraceae	NA	uncultured bacterium	Col_0_dde2	*dde2*	Verrucomicrobiae;NA;OTU_5687
OTU_57	-1.241111919	Bacilli	Paenibacillaceae	Paenibacillus	uncultured bacterium	Col_0_fad7_1fad8_1	*fad7_1fad8_1*	Bacilli;Paenibacillus;OTU_57
OTU_58	-1.050292513	Thermoleophilia	67-14	NA	uncultured bacterium	Col_0_fad7_1fad8_1	*fad7_1fad8_1*	Thermoleophilia;NA;OTU_58
OTU_597	6.362220292	Alphaproteobacteria	Rhizobiaceae	ANP-Rhizobium	NA	Col_0_fad7_1fad8_1	*fad7_1fad8_1*	Alphaproteobacteria;ANP-Rhizobium;OTU_597
OTU_60	1.170450311	Alphaproteobacteria	Devosiaceae	Devosia	uncultured bacterium	Col_0_fad7_1fad8_1	*fad7_1fad8_1*	Alphaproteobacteria;Devosia;OTU_60
OTU_6428	7.765034808	Gammaproteobacteria	Burkholderiaceae	Variovorax	NA	Col_0_dde2	*dde2*	Gammaproteobacteria;Variovorax;OTU_6428
OTU_6428	6.217374226	Gammaproteobacteria	Burkholderiaceae	Variovorax	NA	Col_0_etr1_3	*etr1_3*	Gammaproteobacteria;Variovorax;OTU_6428
OTU_643	2.126918974	Bacilli	Paenibacillaceae	Paenibacillus	NA	Col_0_dde2	*dde2*	Bacilli;Paenibacillus;OTU_643

OTU_667	-4.415235276	Bacteroidia	Flavobacteriaceae	Flavobacterium	NA	Ler_0_aba3_2	*aba3_2*	Bacteroidia;Flavobacterium;OTU_667
OTU_667	-5.392259739	Bacteroidia	Flavobacteriaceae	Flavobacterium	NA	Col_0_etr1_3	*etr1_3*	Bacteroidia;Flavobacterium;OTU_667
OTU_667	-5.041585444	Bacteroidia	Flavobacteriaceae	Flavobacterium	NA	Col_0_35S_ERF	*35S_ERF*	Bacteroidia;Flavobacterium;OTU_667
OTU_667	-5.465240875	Bacteroidia	Flavobacteriaceae	Flavobacterium	NA	Col_0_fad7_1fad8_1	*fad7_1fad8_1*	Bacteroidia;Flavobacterium;OTU_667
OTU_68	-1.577464819	Thermoleophilia	uncultured	NA	uncultured bacterium	Col_0_etr1_3	*etr1_3*	Thermoleophilia;NA;OTU_68
OTU_7	0.888027473	Alphaproteobacteria	Xanthobacteraceae	Bradyrhizobium	Ambiguous_taxa	Ler_0_aba3_2	*aba3_2*	Alphaproteobacteria;Bradyrhizobium;OTU_7
OTU_70	-1.514711719	Actinobacteria	Nocardioidaceae	Nocardioides	uncultured bacterium	Col_0_etr1_3	*etr1_3*	Actinobacteria;Nocardioides;OTU_70
OTU_70	-1.479596224	Actinobacteria	Nocardioidaceae	Nocardioides	uncultured bacterium	Col_0_fad7_1fad8_1	*fad7_1fad8_1*	Actinobacteria;Nocardioides;OTU_70
OTU_71	0.599226748	Alphaproteobacteria	Xanthobacteraceae	Bradyrhizobium	Ambiguous_taxa	Col_0_fad7_1fad8_1	*fad7_1fad8_1*	Alphaproteobacteria;Bradyrhizobium;OTU_71
OTU_72	-1.972888422	Actinobacteria	Geodermatophilaceae	Blastococcus	uncultured bacterium	Col_0_etr1_3	*etr1_3*	Actinobacteria;Blastococcus;OTU_72
OTU_7238	-5.025754328	Verrucomicrobiae	Verrucomicrobiaceae	Prosthecobacter	uncultured bacterium	Col_0_etr1_3	*etr1_3*	Verrucomicrobiae;Prosthecobacter;OTU_7238
OTU_7238	-5.758505712	Verrucomicrobiae	Verrucomicrobiaceae	Prosthecobacter	uncultured bacterium	Col_0_fad7_1fad8_1	*fad7_1fad8_1*	Verrucomicrobiae;Prosthecobacter;OTU_7238
OTU_774	6.385025227	Bacteroidia	Weeksellaceae	Chryseobacterium	Ambiguous_taxa	Col_0_dde2	*dde2*	Bacteroidia;Chryseobacterium;OTU_774
OTU_79	1.914027918	Verrucomicrobiae	Chthoniobacteraceae	Cand. Udaeobacter	NA	Col_0_etr1_3	*etr1_3*	Verrucomicrobiae;Cand. Udaeobacter;OTU_79
OTU_806	-1.67116143	Thermoleophilia	Solirubrobacteraceae	Solirubrobacter	uncultured bacterium	Col_0_fad7_1fad8_1	*fad7_1fad8_1*	Thermoleophilia;Solirubrobacter;OTU_806
OTU_809	4.427161151	Verrucomicrobiae	Chthoniobacteraceae	Chthoniobacter	NA	Col_0_dde2	*dde2*	Verrucomicrobiae;Chthoniobacter;OTU_809
OTU_81	2.039259394	Verrucomicrobiae	Chthoniobacteraceae	Cand. Udaeobacter	uncultured Verrucomicrobia bacterium	Col_0_etr1_3	*etr1_3*	Verrucomicrobiae;Cand. Udaeobacter;OTU_81
OTU_82	-1.627744635	Actinobacteria	Nocardioidaceae	Marmoricola	Ambiguous_taxa	Col_0_fad7_1fad8_1	*fad7_1fad8_1*	Actinobacteria;Marmoricola;OTU_82
OTU_854	3.512076714	Planctomycetacia	Gemmataceae	NA	uncultured bacterium	Col_0_etr1_3	*etr1_3*	Planctomycetacia;NA;OTU_854
OTU_874	-5.272260217	Gammaproteobacteria	Xanthomonadaceae	Stenotrophomonas	Stenotrophomonas maltophilia	Col_0_fad7_1fad8_1	*fad7_1fad8_1*	Gammaproteobacteria;Stenotrophomonas;OTU_874
OTU_878	5.54480128	Gammaproteobacteria	Burkholderiaceae	Variovorax	Ambiguous_taxa	Col_0_dde2	*dde2*	Gammaproteobacteria;Variovorax;OTU_878
OTU_94	5.102082543	Alphaproteobacteria	Sphingomonadaceae	Sphingopyxis	NA	Col_0_dde2	*dde2*	Alphaproteobacteria;Sphingopyxis;OTU_94
OTU_97	-2.54648125	Actinobacteria	Nocardioidaceae	Nocardioides	uncultured bacterium	Col_0_etr1_3	*etr1_3*	Actinobacteria;Nocardioides;OTU_97
OTU_97	-1.406715336	Actinobacteria	Nocardioidaceae	Nocardioides	uncultured bacterium	Col_0_fad7_1fad8_1	*fad7_1fad8_1*	Actinobacteria;Nocardioides;OTU_97
OTU_9861	-1.712685781	Verrucomicrobiae	Chthoniobacteraceae	Cand. Udaeobacter	uncultured Xiphinematobacteriaceae bacterium	Ler_0_aba3_2	*aba3_2*	Verrucomicrobiae;Cand. Udaeobacter;OTU_9861
OTU_9861	1.558866768	Verrucomicrobiae	Chthoniobacteraceae	Cand. Udaeobacter	uncultured Xiphinematobacteriaceae bacterium	Col_0_etr1_3	*etr1_3*	Verrucomicrobiae;Cand. Udaeobacter;OTU_9861

Table S10: Differentially abundant root fungal OTUs of Col_0 and GLS mutants as determined using DESeq2.

otu	FoldChange	Phylum	Class	Family	Genus	Species	WT_Mutant	Mutant	Taxonomy_OTU
OTU_100	-7.224048037	Ascomycota	Dothideomycetes	unidentified	NA	unidentified	Col_0_cyp79B2cyp79B3	*cyp79B2cyp79B3*	Dothideomycetes;NA;OTU_100
OTU_100	-6.020535038	Ascomycota	Dothideomycetes	unidentified	NA	unidentified	Col_0_myb51	*myb51*	Dothideomycetes;NA;OTU_100
OTU_100	-6.602500464	Ascomycota	Dothideomycetes	unidentified	NA	unidentified	Col_0_gsm1_1	*gsm1_1*	Dothideomycetes;NA;OTU_100
OTU_100	-7.159314897	Ascomycota	Dothideomycetes	unidentified	NA	unidentified	Col_0_TU3	*TU3*	Dothideomycetes;NA;OTU_100
OTU_101	8.104628594	Basidiomycota	Tremellomycetes	Trichosporonaceae	Apiotrichum	NA	Col_0_Pen3_1NahG	*Pen3_1NahG*	Tremellomycetes;Apiotrichum;OTU_101
OTU_111	-6.362379572	Ascomycota	Sordariomycetes	Nectriaceae	Fusarium	NA	Col_0_Pen3_1NahG	*Pen3_1NahG*	Sordariomycetes;Fusarium;OTU_111
OTU_120	8.50745825	Ascomycota	Leotiomycetes	unidentified	NA	unidentified	Col_0_Pen3_1NahG	*Pen3_1NahG*	Leotiomycetes;NA;OTU_120
OTU_125	27.00414729	Basidiomycota	Agaricomycetes	Entolomataceae	Clitopilus	unidentified	Col_0_Pen3_1NahG	*Pen3_1NahG*	Agaricomycetes;Clitopilus;OTU_125
OTU_136	6.32554767	Ascomycota	Leotiomycetes	Leotiaceae	Neobulgaria	NA	Col_0_Pen3_1NahG	*Pen3_1NahG*	Leotiomycetes;Neobulgaria;OTU_136
OTU_139	8.021176705	Ascomycota	Eurotiomycetes	unidentified	NA	unidentified	Col_0_gsm1_1	*gsm1_1*	Eurotiomycetes;NA;OTU_139
OTU_139	5.641735042	Ascomycota	Eurotiomycetes	unidentified	NA	unidentified	Col_0_Pen3_1NahG	*Pen3_1NahG*	Eurotiomycetes;NA;OTU_139
OTU_16	-2.723717198	Ascomycota	Dothideomycetes	Didymosphaeriaceae	Paraphaeosphaeria	NA	Col_0_TU3	*TU3*	Dothideomycetes;Paraphaeosphaeria;OTU_16
OTU_171	10.70600693	Basidiomycota	Agaricomycetes	Cantharellales_fam_Incertae_sedis	Sistotrema	NA	Col_0_Pen3_1NahG	*Pen3_1NahG*	Agaricomycetes;Sistotrema;OTU_171
OTU_18	2.503726554	Ascomycota	Sordariomycetes	Bionectriaceae	Clonostachys	Clonostachys_rosea	Col_0_TU3	*TU3*	Sordariomycetes;Clonostachys;OTU_18
OTU_182	-6.426614176	Ascomycota	Sordariomycetes	unidentified	NA	unidentified	Col_0_TU3	*TU3*	Sordariomycetes;NA;OTU_182
OTU_186	9.778297109	Ascomycota	Eurotiomycetes	Herpotrichiellaceae	Exophiala	NA	Col_0_gsm1_1	*gsm1_1*	Eurotiomycetes;Exophiala;OTU_186
OTU_186	7.460760485	Ascomycota	Eurotiomycetes	Herpotrichiellaceae	Exophiala	NA	Col_0_TU3	*TU3*	Eurotiomycetes;Exophiala;OTU_186
OTU_193	7.965431551	Ascomycota	Leotiomycetes	unidentified	NA	unidentified	Col_0_cyp79B2cyp79B3	*cyp79B2cyp79B3*	Leotiomycetes;NA;OTU_193
OTU_193	8.174227241	Ascomycota	Leotiomycetes	unidentified	NA	unidentified	Col_0_Pen3_1NahG	*Pen3_1NahG*	Leotiomycetes;NA;OTU_193
OTU_194	7.352115175	Ascomycota	Leotiomycetes	unidentified	NA	unidentified	Col_0_TU3	*TU3*	Leotiomycetes;NA;OTU_194
OTU_244	7.793107687	Ascomycota	Eurotiomycetes	Herpotrichiellaceae	Exophiala	NA	Col_0_myb51	*myb51*	Eurotiomycetes;Exophiala;OTU_244
OTU_244	8.280453242	Ascomycota	Eurotiomycetes	Herpotrichiellaceae	Exophiala	NA	Col_0_gsm1_1	*gsm1_1*	Eurotiomycetes;Exophiala;OTU_244
OTU_25	3.092406808	Ascomycota	Sordariomycetes	Nectriaceae	Fusarium	NA	Col_0_myb51	*myb51*	Sordariomycetes;Fusarium;OTU_25
OTU_25	3.521089858	Ascomycota	Sordariomycetes	Nectriaceae	Fusarium	NA	Col_0_TU3	*TU3*	Sordariomycetes;Fusarium;OTU_25
OTU_252	7.067751758	Ascomycota	Leotiomycetes	Pseudeurotiaceae	NA	NA	Col_0_TU3	*TU3*	Leotiomycetes;NA;OTU_252
OTU_26	-6.856673713	Basidiomycota	Tremellomycetes	Piskurozymaceae	Solicoccozyma	Solicoccozyma_terricola	Col_0_cyp79B2cyp79B3	*cyp79B2cyp79B3*	Tremellomycetes;Solicoccozyma;OTU_26
OTU_27	-6.989841775	Ascomycota	Sordariomycetes	Magnaporthaceae	Falciphora	NA	Col_0_cyp79B2cyp79B3	*cyp79B2cyp79B3*	Sordariomycetes;Falciphora;OTU_27
OTU_270	-6.476351049	Ascomycota	Eurotiomycetes	Herpotrichiellaceae	NA	unidentified	Col_0_cyp79B2cyp79B3	*cyp79B2cyp79B3*	Eurotiomycetes;NA;OTU_270
OTU_270	-6.094106803	Ascomycota	Eurotiomycetes	Herpotrichiellaceae	NA	unidentified	Col_0_myb51	*myb51*	Eurotiomycetes;NA;OTU_270

OTU_270	-6.336274812	Ascomycota	Eurotiomycetes	Herpotrichiellaceae	NA	unidentified	Col_0_TU3	*TU3*	Eurotiomycetes;NA;OTU_270
OTU_28	8.997176222	Ascomycota	Dothideomycetes	unidentified	NA	unidentified	Col_0_gsm1_1	*gsm1_1*	Dothideomycetes;NA;OTU_28
OTU_280	8.494216657	Ascomycota	Eurotiomycetes	Herpotrichiellaceae	Cladophialophora	unidentified	Col_0_cyp79B2cyp79B3	*cyp79B2cyp79B3*	Eurotiomycetes;Cladophialophora;OTU_280
OTU_281	7.737235207	Ascomycota	Dothideomycetes	unidentified	NA	unidentified	Col_0_Pen3_1NahG	*Pen3_1NahG*	Dothideomycetes;NA;OTU_281
OTU_301	8.316215013	Ascomycota	Dothideomycetes	NA	NA	NA	Col_0_gsm1_1	*gsm1_1*	Dothideomycetes;NA;OTU_301
OTU_311	-22.79707674	Basidiomycota	Microbotryomycetes	Sporidiobolaceae	Sporobolomyces	NA	Col_0_cyp79B2cyp79B3	*cyp79B2cyp79B3*	Microbotryomycetes;Sporobolomyces;OTU_311
OTU_311	-23.02518505	Basidiomycota	Microbotryomycetes	Sporidiobolaceae	Sporobolomyces	NA	Col_0_myb51	*myb51*	Microbotryomycetes;Sporobolomyces;OTU_311
OTU_311	-22.55976342	Basidiomycota	Microbotryomycetes	Sporidiobolaceae	Sporobolomyces	NA	Col_0_gsm1_1	*gsm1_1*	Microbotryomycetes;Sporobolomyces;OTU_311
OTU_311	-23.95758871	Basidiomycota	Microbotryomycetes	Sporidiobolaceae	Sporobolomyces	NA	Col_0_TU3	*TU3*	Microbotryomycetes;Sporobolomyces;OTU_311
OTU_311	-25.65827059	Basidiomycota	Microbotryomycetes	Sporidiobolaceae	Sporobolomyces	NA	Col_0_Pen3_1NahG	*Pen3_1NahG*	Microbotryomycetes;Sporobolomyces;OTU_311
OTU_333	8.019538913	Ascomycota	Dothideomycetes	unidentified	NA	unidentified	Col_0_cyp79B2cyp79B3	*cyp79B2cyp79B3*	Dothideomycetes;NA;OTU_333
OTU_355	10.28022705	Ascomycota	Leotiomycetes	Helotiaceae	Hymenoscyphus	Hymenoscyphus_albidus	Col_0_myb51	*myb51*	Leotiomycetes;Hymenoscyphus;OTU_355
OTU_382	7.554816276	Basidiomycota	Agaricomycetes	Entolomataceae	Entoloma	unidentified	Col_0_Pen3_1NahG	*Pen3_1NahG*	Agaricomycetes;Entoloma;OTU_382
OTU_4	-11.7593003	Ascomycota	Sordariomycetes	Hypocreales_fam_Incertae_sedis	Emericellopsis	NA	Col_0_cyp79B2cyp79B3	*cyp79B2cyp79B3*	Sordariomycetes;Emericellopsis;OTU_4
OTU_4	-10.80017259	Ascomycota	Sordariomycetes	Hypocreales_fam_Incertae_sedis	Emericellopsis	NA	Col_0_myb51	*myb51*	Sordariomycetes;Emericellopsis;OTU_4
OTU_411	7.008670357	Ascomycota	Sordariomycetes	Coniochaetaceae	NA	NA	Col_0_Pen3_1NahG	*Pen3_1NahG*	Sordariomycetes;NA;OTU_411
OTU_42	-5.589118733	Ascomycota	Sordariomycetes	Glomerellaceae	Colletotrichum	NA	Col_0_gsm1_1	*gsm1_1*	Sordariomycetes;Colletotrichum;OTU_42
OTU_43	-10.53850192	Ascomycota	Sordariomycetes	Hypocreales_fam_Incertae_sedis	Emericellopsis	NA	Col_0_gsm1_1	*gsm1_1*	Sordariomycetes;Emericellopsis;OTU_43
OTU_44	-12.09450331	Ascomycota	Sordariomycetes	Hypocreales_fam_Incertae_sedis	Emericellopsis	NA	Col_0_TU3	*TU3*	Sordariomycetes;Emericellopsis;OTU_44
OTU_482	7.959349957	Basidiomycota	Agaricomycetes	Psathyrellaceae	Coprinellus	NA	Col_0_gsm1_1	*gsm1_1*	Agaricomycetes;Coprinellus;OTU_482
OTU_504	8.531258647	Ascomycota	Leotiomycetes	Helotiales_fam_Incertae_sedis	Cadophora	NA	Col_0_Pen3_1NahG	*Pen3_1NahG*	Leotiomycetes;Cadophora;OTU_504
OTU_53	-8.073406249	Ascomycota	Pezizomycetes	Pyronemataceae	NA	unidentified	Col_0_cyp79B2cyp79B3	*cyp79B2cyp79B3*	Pezizomycetes;NA;OTU_53
OTU_53	-7.87021594	Ascomycota	Pezizomycetes	Pyronemataceae	NA	unidentified	Col_0_myb51	*myb51*	Pezizomycetes;NA;OTU_53
OTU_53	-7.558157186	Ascomycota	Pezizomycetes	Pyronemataceae	NA	unidentified	Col_0_gsm1_1	*gsm1_1*	Pezizomycetes;NA;OTU_53
OTU_54	-4.864588699	Ascomycota	Sordariomycetes	NA	NA	NA	Col_0_TU3	*TU3*	Sordariomycetes;NA;OTU_54
OTU_55	4.521480462	Ascomycota	Sordariomycetes	Bionectriaceae	Clonostachys	NA	Col_0_TU3	*TU3*	Sordariomycetes;Clonostachys;OTU_55
OTU_56	-7.702258662	Basidiomycota	Tremellomycetes	Tetragoniomycetaceae	Tetragoniomyces	Tetragoniomyces_uliginosus	Col_0_cyp79B2cyp79B3	*cyp79B2cyp79B3*	Tremellomycetes;Tetragoniomyces;OTU_56
OTU_560	23.1456711	Basidiomycota	Tremellomycetes	Trichosporonaceae	Cutaneotrichosporon	NA	Col_0_cyp79B2cyp79B3	*cyp79B2cyp79B3*	Tremellomycetes;Cutaneotrichosporon;OTU_560
OTU_594	10.32802544	Ascomycota	Eurotiomycetes	Herpotrichiellaceae	Exophiala	NA	Col_0_gsm1_1	*gsm1_1*	Eurotiomycetes;Exophiala;OTU_594
OTU_71	7.381295707	Ascomycota	Leotiomycetes	Helotiaceae	Hymenoscyphus	NA	Col_0_TU3	*TU3*	Leotiomycetes;Hymenoscyphus;OTU_71

OTU_79	-6.746725952	Ascomycota	Ascomycota (Unknown Class)	NA	NA	NA	Col_0_cyp79B2cyp79B3	*cyp79B2cyp79B3*	Ascomycota (Unknown Class);NA;OTU_79
OTU_79	-6.140614984	Ascomycota	Ascomycota (Unknown Class)	NA	NA	NA	Col_0_myb51	*myb51*	Ascomycota (Unknown Class);NA;OTU_79
OTU_79	-6.148299027	Ascomycota	Ascomycota (Unknown Class)	NA	NA	NA	Col_0_gsm1_1	*gsm1_1*	Ascomycota (Unknown Class);NA;OTU_79

Table S11: Differentially abundant root fungal OTUs of Col_0 and Ler_0 and FLV mutants as determined using DESeq2.

otu	FoldChange	Phylum	Class	Family	Genus	Species	WT_Mutant	Mutant	Taxonomy_OTU
OTU_100	7.504574699	Ascomycota	Dothideomycetes	unidentified	NA	unidentified	Ler_0_tt5	*tt5*	Dothideomycetes;NA;OTU_100
OTU_100	-7.239164302	Ascomycota	Dothideomycetes	unidentified	NA	unidentified	Col_0_pap1_D	*pap1_D*	Dothideomycetes;NA;OTU_100
OTU_101	-7.921424937	Basidiomycota	Tremellomycetes	Trichosporonaceae	Apiotrichum	NA	Ler_0_tt5	*tt5*	Tremellomycetes;Apiotrichum;OTU_101
OTU_101	-6.681159279	Basidiomycota	Tremellomycetes	Trichosporonaceae	Apiotrichum	NA	Ler_0_tt3_1tt5_1	*tt3_1tt5_1*	Tremellomycetes;Apiotrichum;OTU_101
OTU_108	8.177646591	Ascomycota	Sordariomycetes	Hypocreaceae	Trichoderma	NA	Ler_0_tt7_7	*tt7_7*	Sordariomycetes;Trichoderma;OTU_108
OTU_108	7.212444929	Ascomycota	Sordariomycetes	Hypocreaceae	Trichoderma	NA	Ler_0_tt5	*tt5*	Sordariomycetes;Trichoderma;OTU_108
OTU_112	7.87917308	Ascomycota	Sordariomycetes	Clavicipitaceae	NA	NA	Ler_0_tt5	*tt5*	Sordariomycetes;NA;OTU_112
OTU_120	8.213518001	Ascomycota	Leotiomycetes	unidentified	NA	unidentified	Col_0_pap1_D	*pap1_D*	Leotiomycetes;NA;OTU_120
OTU_13	2.946962204	Mortierellomycota	Mortierellomycetes	Mortierellaceae	Mortierella	NA	Ler_0_tt5	*tt5*	Mortierellomycetes;Mortierella;OTU_13
OTU_136	-7.114027276	Ascomycota	Leotiomycetes	Leotiaceae	Neobulgaria	NA	Ler_0_tt5	*tt5*	Leotiomycetes;Neobulgaria;OTU_136
OTU_139	8.143440055	Ascomycota	Eurotiomycetes	unidentified	NA	unidentified	Ler_0_tt3	*tt3*	Eurotiomycetes;NA;OTU_139
OTU_139	8.143946282	Ascomycota	Eurotiomycetes	unidentified	NA	unidentified	Ler_0_tt5	*tt5*	Eurotiomycetes;NA;OTU_139
OTU_139	7.434121389	Ascomycota	Eurotiomycetes	unidentified	NA	unidentified	Ler_0_tt3_1tt5_1	*tt3_1tt5_1*	Eurotiomycetes;NA;OTU_139
OTU_143	6.603545949	Ascomycota	Sordariomycetes	Clavicipitaceae	NA	unidentified	Ler_0_tt7_7	*tt7_7*	Sordariomycetes;NA;OTU_143
OTU_144	-9.648528755	Basidiomycota	Agaricomycetes	Ceratobasidiaceae	NA	NA	Ler_0_tt7_7	*tt7_7*	Agaricomycetes;NA;OTU_144
OTU_144	-8.926474574	Basidiomycota	Agaricomycetes	Ceratobasidiaceae	NA	NA	Ler_0_tt3	*tt3*	Agaricomycetes;NA;OTU_144
OTU_144	-9.389756634	Basidiomycota	Agaricomycetes	Ceratobasidiaceae	NA	NA	Ler_0_tt5	*tt5*	Agaricomycetes;NA;OTU_144
OTU_144	-7.403201801	Basidiomycota	Agaricomycetes	Ceratobasidiaceae	NA	NA	Ler_0_tt3_1tt5_1	*tt3_1tt5_1*	Agaricomycetes;NA;OTU_144
OTU_149	21.35585122	Ascomycota	Dothideomycetes	unidentified	NA	unidentified	Ler_0_tt7_7	*tt7_7*	Dothideomycetes;NA;OTU_149
OTU_15	-3.527664177	Ascomycota	Sordariomycetes	NA	NA	NA	Ler_0_tt7_7	*tt7_7*	Sordariomycetes;NA;OTU_15
OTU_158	7.878985515	Ascomycota	Leotiomycetes	Helotiaceae	Hymenoscyphus	NA	Ler_0_tt7_7	*tt7_7*	Leotiomycetes;Hymenoscyphus;OTU_158
OTU_16	3.222652392	Ascomycota	Dothideomycetes	Didymosphaeriaceae	Paraphaeosphaeria	NA	Ler_0_tt3	*tt3*	Dothideomycetes;Paraphaeosphaeria;OTU_16
OTU_161	3.484884164	Ascomycota	Dothideomycetes	Didymosphaeriaceae	Paraphaeosphaeria	NA	Ler_0_tt5	*tt5*	Dothideomycetes;Paraphaeosphaeria;OTU_161

OTU_162	-7.17060987	Ascomycota	Dothideomycetes	NA	NA	NA	Ler_0_tt3	*tt3*	Dothideomycetes;NA;OTU_162
OTU_162	-7.816963548	Ascomycota	Dothideomycetes	NA	NA	NA	Ler_0_tt5	*tt5*	Dothideomycetes;NA;OTU_162
OTU_175	-7.329790935	Basidiomycota	Tremellomycetes	Holtermanniales_fam_Incertae_sedis	Holtermanniella	Holtermanniella_takashimae	Ler_0_tt3	*tt3*	Tremellomycetes;Holtermanniella;OTU_175
OTU_175	-7.978128683	Basidiomycota	Tremellomycetes	Holtermanniales_fam_Incertae_sedis	Holtermanniella	Holtermanniella_takashimae	Ler_0_tt5	*tt5*	Tremellomycetes;Holtermanniella;OTU_175
OTU_18	3.838093048	Ascomycota	Sordariomycetes	Bionectriaceae	Clonostachys	Clonostachys_rosea	Ler_0_tt3	*tt3*	Sordariomycetes;Clonostachys;OTU_18
OTU_18	5.449163879	Ascomycota	Sordariomycetes	Bionectriaceae	Clonostachys	Clonostachys_rosea	Ler_0_tt5	*tt5*	Sordariomycetes;Clonostachys;OTU_18
OTU_18	-6.50283525	Ascomycota	Sordariomycetes	unidentified	NA	unidentified	Col_0_pap1_D	*pap1_D*	Sordariomycetes;NA;OTU_18
OTU_18	3.523682321	Ascomycota	Sordariomycetes	Bionectriaceae	Clonostachys	Clonostachys_rosea	Col_0_pap1_D	*pap1_D*	Sordariomycetes;Clonostachys;OTU_18
OTU_185	6.523850525	Mortierellomycota	Mortierellomycetes	Mortierellaceae	Mortierella	Mortierella_sarnyensis	Col_0_pap1_D	*pap1_D*	Mortierellomycetes;Mortierella;OTU_185
OTU_19	4.439279853	Ascomycota	Dothideomycetes	unidentified	NA	unidentified	Ler_0_tt7_7	*tt7_7*	Dothideomycetes;NA;OTU_19
OTU_192	9.623519229	Ascomycota	Dothideomycetes	Tubeufiaceae	NA	unidentified	Col_0_pap1_D	*pap1_D*	Dothideomycetes;NA;OTU_192
OTU_193	8.113579353	Ascomycota	Leotiomycetes	unidentified	NA	unidentified	Col_0_pap1_D	*pap1_D*	Leotiomycetes;NA;OTU_193
OTU_194	-7.67308984	Ascomycota	Leotiomycetes	unidentified	NA	unidentified	Ler_0_tt5	*tt5*	Leotiomycetes;NA;OTU_194
OTU_194	6.939888845	Ascomycota	Leotiomycetes	unidentified	NA	unidentified	Col_0_pap1_D	*pap1_D*	Leotiomycetes;NA;OTU_1941
OTU_2	3.416073064	Ascomycota	Sordariomycetes	NA	NA	NA	Ler_0_tt7_7	*tt7_7*	Sordariomycetes;NA;OTU_2
OTU_202	-7.64465726	Basidiomycota	Agaricomycetes	Psathyrellaceae	Psathyrella	NA	Ler_0_tt7_7	*tt7_7*	Agaricomycetes;Psathyrella;OTU_202
OTU_202	-7.249520882	Basidiomycota	Agaricomycetes	Psathyrellaceae	Psathyrella	NA	Ler_0_tt3	*tt3*	Agaricomycetes;Psathyrella;OTU_202
OTU_21	-6.078779002	Ascomycota	Sordariomycetes	Lasiosphaeriaceae	NA	NA	Ler_0_tt3_1tt5_1	*tt3_1tt5_1*	Sordariomycetes;NA;OTU_21
OTU_212	-7.379448187	Basidiomycota	Tremellomycetes	Trichosporonaceae	Apiotrichum	NA	Ler_0_tt7_7	*tt7_7*	Tremellomycetes;Apiotrichum;OTU_212
OTU_212	-7.673812139	Basidiomycota	Tremellomycetes	Trichosporonaceae	Apiotrichum	NA	Ler_0_tt5	*tt5*	Tremellomycetes;Apiotrichum;OTU_212
OTU_219	-7.213033222	Ascomycota	Dothideomycetes	Sporormiaceae	Westerdykella	NA	Ler_0_tt7_7	*tt7_7*	Dothideomycetes;Westerdykella;OTU_219
OTU_219	-7.208992094	Ascomycota	Dothideomycetes	Sporormiaceae	Westerdykella	NA	Ler_0_tt5	*tt5*	Dothideomycetes;Westerdykella;OTU_219
OTU_22	3.326434838	Ascomycota	Sordariomycetes	NA	NA	NA	Ler_0_tt5	*tt5*	Sordariomycetes;NA;OTU_22
OTU_240	-23.11190232	Ascomycota	Sordariomycetes	Chaetomiaceae	Zopfiella	unidentified	Ler_0_tt7_7	*tt7_7*	Sordariomycetes;Zopfiella;OTU_240
OTU_275	7.24126511	Ascomycota	Ascomycota (Unknown Class)	unidentified	NA	unidentified	Col_0_pap1_D	*pap1_D*	Ascomycota (Unknown Class);NA;OTU_275
OTU_291	-6.389045442	Ascomycota	Leotiomycetes	Pseudeurotiaceae	Pseudogymnoascus	NA	Col_0_pap1_D	*pap1_D*	Leotiomycetes;Pseudogymnoascus;OTU_291
OTU_295	-7.404072901	Ascomycota	Sordariomycetes	NA	NA	NA	Ler_0_tt7_7	*tt7_7*	Sordariomycetes;NA;OTU_295
OTU_295	-7.427866761	Ascomycota	Sordariomycetes	NA	NA	NA	Ler_0_tt5	*tt5*	Sordariomycetes;NA;OTU_295
OTU_308	-7.143617052	Basidiomycota	Basidiomycota (Unknown Class)	NA	NA	NA	Ler_0_tt7_7	*tt7_7*	Basidiomycota (Unknown Class);NA;OTU_308
OTU_308	-7.380831463	Basidiomycota	Basidiomycota (Unknown Class)	NA	NA	NA	Ler_0_tt5	*tt5*	Basidiomycota (Unknown Class);NA;OTU_308
OTU_31	-4.695872277	Ascomycota	Leotiomycetes	Helotiales_fam_Incertae_sedis	NA	NA	Ler_0_tt7_7	*tt7_7*	Leotiomycetes;NA;OTU_31

OTU_311	-24.29640394	Basidiomycota	Microbotryomycetes	Sporidiobolaceae	Sporobolomyces	NA	Col_0_pap1_D	*pap1_D*	Microbotryomycetes;Sporobolomyces;OTU_311
OTU_312	-5.126665422	Ascomycota	Leotiomycetes	Helotiales_fam_Incertae_sedis	NA	NA	Ler_0_tt3	*tt3*	Leotiomycetes;NA;OTU_312
OTU_34	-7.927300026	Ascomycota	Leotiomycetes	NA	NA	NA	Ler_0_tt5	*tt5*	Leotiomycetes;NA;OTU_34
OTU_350	6.921965834	Ascomycota	Dothideomycetes	Pleosporaceae	Alternaria	NA	Ler_0_tt7_7	*tt7_7*	Dothideomycetes;Alternaria;OTU_350
OTU_4	10.52284637	Ascomycota	Sordariomycetes	Hypocreales_fam_Incertae_sedis	Emericellopsis	NA	Ler_0_tt7_7	*tt7_7*	Sordariomycetes;Emericellopsis;OTU_4
OTU_4	10.30558331	Ascomycota	Sordariomycetes	Hypocreales_fam_Incertae_sedis	Emericellopsis	NA	Ler_0_tt5	*tt5*	Sordariomycetes;Emericellopsis;OTU_4
OTU_42	5.014411696	Ascomycota	Sordariomycetes	Glomerellaceae	Colletotrichum	NA	Ler_0_tt3	*tt3*	Sordariomycetes;Colletotrichum;OTU_42
OTU_4	-11.58972356	Ascomycota	Sordariomycetes	Hypocreales_fam_Incertae_sedis	Emericellopsis	NA	Col_0_pap1_D	*pap1_D*	Sordariomycetes;Emericellopsis;OTU_4
OTU_510	8.830940074	Basidiomycota	Agaricomycetes	Podoscyphaceae	Cotylidia	Cotylidia_undulata	Col_0_pap1_D	*pap1_D*	Agaricomycetes;Cotylidia;OTU_510
OTU_53	-2.890786682	Ascomycota	Pezizomycetes	Pyronemataceae	NA	unidentified	Col_0_pap1_D	*pap1_D*	Pezizomycetes;NA;OTU_53
OTU_568	6.866834602	Ascomycota	Eurotiomycetes	Herpotrichiellaceae	NA	unidentified	Col_0_pap1_D	*pap1_D*	Eurotiomycetes;NA;OTU_568
OTU_594	8.468717385	Ascomycota	Eurotiomycetes	Herpotrichiellaceae	Exophiala	NA	Ler_0_tt3_1tt5_1	*tt3_1tt5_1*	Eurotiomycetes;Exophiala;OTU_594
OTU_62	9.295681979	Ascomycota	Eurotiomycetes	Herpotrichiellaceae	Exophiala	NA	Ler_0_tt3_1tt5_1	*tt3_1tt5_1*	Eurotiomycetes;Exophiala;OTU_62
OTU_681	7.559506803	Olpidiomycota	Olpidiomycetes	Olpidiaceae	Olpidium	Olpidium_brassicae	Ler_0_tt3	*tt3*	Olpidiomycetes;Olpidium;OTU_681
OTU_73	-4.784809598	Basidiomycota	Agaricomycetes	Cantharellales_fam_Incertae_sedis	Minimedusa	Minimedusa_polyspora	Ler_0_tt5	*tt5*	Agaricomycetes;Minimedusa;OTU_73
OTU_73	-5.207134918	Basidiomycota	Agaricomycetes	Cantharellales_fam_Incertae_sedis	Minimedusa	Minimedusa_polyspora	Ler_0_tt3_1tt5_1	*tt3_1tt5_1*	Agaricomycetes;Minimedusa;OTU_73
OTU_97	-7.837020982	Ascomycota	Sordariomycetes	Lasiosphaeriaceae	Apodus	NA	Ler_0_tt3	*tt3*	Sordariomycetes;Apodus;OTU_97

Table S12: Differentially abundant root fungal OTUs of Col_0 and Ler_0 and DSMs mutants as determined using DESeq2.

**otu**	**FoldChange**	**Phylum**	**Class**	**Family**	**Genus**	**Species**	**WT_Mutant**	**Mutant**	**Taxonomy_OTU**
OTU_10	1.882103053	Ascomycota	Eurotiomycetes	Herpotrichiellaceae	Exophiala	Exophiala_equina	Col_0_dde2	*dde2*	Eurotiomycetes;Exophiala;OTU_10
OTU_100	-7.495754075	Ascomycota	Dothideomycetes	unidentified	NA	unidentified	Col_0_dde2	*dde2*	Dothideomycetes;NA;OTU_100
OTU_100	6.425878972	Ascomycota	Dothideomycetes	unidentified	NA	unidentified	Ler_0_aba3_2	*aba3_2*	Dothideomycetes;NA;OTU_100
OTU_100	-7.006268931	Ascomycota	Dothideomycetes	unidentified	NA	unidentified	Col_0_etr1_3	*etr1_3*	Dothideomycetes;NA;OTU_100
OTU_101	28.46061325	Basidiomycota	Tremellomycetes	Trichosporonaceae	Apiotrichum	NA	Col_0_etr1_3	*etr1_3*	Tremellomycetes;Apiotrichum;OTU_101
OTU_101	8.202767347	Basidiomycota	Tremellomycetes	Trichosporonaceae	Apiotrichum	NA	Col_0_35S_ERF	*35S_ERF*	Tremellomycetes;Apiotrichum;OTU_101
OTU_102	7.285957569	Ascomycota	Sordariomycetes	Nectriaceae	Cosmospora	NA	Ler_0_aba3_2	*aba3_2*	Sordariomycetes;Cosmospora;OTU_102
OTU_112	8.00748141	Ascomycota	Sordariomycetes	Clavicipitaceae	NA	NA	Ler_0_aba3_2	*aba3_2*	Sordariomycetes;NA;OTU_112
OTU_112	-6.885221005	Ascomycota	Sordariomycetes	Clavicipitaceae	NA	NA	Col_0_35S_ERF	*35S_ERF*	Sordariomycetes;NA;OTU_112

OTU_138	-6.311944529	Mortierellomycota	Mortierellomycetes	Mortierellaceae	Mortierella	NA	Col_0_fad3_2	*fad3_2*	Mortierellomycetes;Mortierella;OTU_138
OTU_16	2.934218836	Ascomycota	Dothideomycetes	Didymosphaeriaceae	Paraphaeosphaeria	NA	Ler_0_aba3_2	*aba3_2*	Dothideomycetes;Paraphaeosphaeria;OTU_16
OTU_169	6.742545838	Ascomycota	Sordariomycetes	Hypocreaceae	Trichoderma	Trichoderma_hamatum	Ler_0_aba3_2	*aba3_2*	Sordariomycetes;Trichoderma;OTU_169
OTU_18	3.780584273	Ascomycota	Sordariomycetes	Bionectriaceae	Clonostachys	Clonostachys_rosea	Col_0_dde2	*dde2*	Sordariomycetes;Clonostachys;OTU_18
OTU_182	-6.451133021	Ascomycota	Sordariomycetes	unidentified	NA	unidentified	Col_0_dde2	*dde2*	Sordariomycetes;NA;OTU_182
OTU_192	9.941350083	Ascomycota	Dothideomycetes	Tubeufiaceae	NA	unidentified	Col_0_fad7_1fad8_1	*fad7_1fad8_1*	Dothideomycetes;NA;OTU_192
OTU_2	4.215495308	Ascomycota	Sordariomycetes	NA	NA	NA	Ler_0_aba3_2	*aba3_2*	Sordariomycetes;NA;OTU_2
OTU_20	-2.373713992	Basidiomycota	Tremellomycetes	Trimorphomycetaceae	Saitozyma	Saitozyma_podzolica	Col_0_etr1_3	*etr1_3*	Tremellomycetes;Saitozyma;OTU_20
OTU_20	-2.607981961	Basidiomycota	Tremellomycetes	Trimorphomycetaceae	Saitozyma	Saitozyma_podzolica	Col_0_35S_ERF	*35S_ERF*	Tremellomycetes;Saitozyma;OTU_20
OTU_270	-5.967881708	Ascomycota	Eurotiomycetes	Herpotrichiellaceae	NA	unidentified	Col_0_etr1_3	*etr1_3*	Eurotiomycetes;NA;OTU_270
OTU_270	-8.058365353	Ascomycota	Eurotiomycetes	Herpotrichiellaceae	NA	unidentified	Col_0_fad7_1fad8_1	*fad7_1fad8_1*	Eurotiomycetes;NA;OTU_270
OTU_28	10.13959186	Ascomycota	Dothideomycetes	unidentified	NA	unidentified	Col_0_dde2	*dde2*	Dothideomycetes;NA;OTU_28
OTU_28	10.18709369	Ascomycota	Dothideomycetes	unidentified	NA	unidentified	Col_0_fad3_2	*fad3_2*	Dothideomycetes;NA;OTU_28
OTU_291	-7.336946384	Ascomycota	Leotiomycetes	Pseudeurotiaceae	Pseudogymnoascus	NA	Col_0_35S_ERF	*35S_ERF*	Leotiomycetes;Pseudogymnoascus;OTU_291
OTU_31	2.733087404	Ascomycota	Leotiomycetes	Helotiales_fam_Incertae_sedis	NA	NA	Col_0_fad7_1fad8_1	*fad7_1fad8_1*	Leotiomycetes;NA;OTU_31
OTU_311	-23.82877559	Basidiomycota	Microbotryomycetes	Sporidiobolaceae	Sporobolomyces	NA	Col_0_dde2	*dde2*	Microbotryomycetes;Sporobolomyces;OTU_311
OTU_311	-22.73712027	Basidiomycota	Microbotryomycetes	Sporidiobolaceae	Sporobolomyces	NA	Col_0_fad3_2	*fad3_2*	Microbotryomycetes;Sporobolomyces;OTU_311
OTU_311	-24.63177062	Basidiomycota	Microbotryomycetes	Sporidiobolaceae	Sporobolomyces	NA	Col_0_fad7_1fad8_1	*fad7_1fad8_1*	Microbotryomycetes;Sporobolomyces;OTU_311
OTU_32	-7.47474356	Ascomycota	Sordariomycetes	Nectriaceae	Fusicolla	NA	Col_0_35S_ERF	*35S_ERF*	Sordariomycetes;Fusicolla;OTU_32
OTU_323	8.102098552	Basidiomycota	Agaricomycetes	NA	NA	NA	Col_0_35S_ERF	*35S_ERF*	Agaricomycetes;NA;OTU_323
OTU_34	7.795898546	Ascomycota	Leotiomycetes	NA	NA	NA	Col_0_35S_ERF	*35S_ERF*	Leotiomycetes;NA;OTU_34
OTU_34	27.04282278	Ascomycota	Leotiomycetes	NA	NA	NA	Col_0_fad3_2	*fad3_2*	Leotiomycetes;NA;OTU_34
OTU_34	7.650526861	Ascomycota	Leotiomycetes	NA	NA	NA	Col_0_fad7_1fad8_1	*fad7_1fad8_1*	Leotiomycetes;NA;OTU_34
OTU_38	-2.676327718	Mortierellomycota	Mortierellomycetes	Mortierellaceae	Mortierella	Mortierella_alpina	Col_0_fad7_1fad8_1	*fad7_1fad8_1*	Mortierellomycetes;Mortierella;OTU_38
OTU_4	-11.53667581	Ascomycota	Sordariomycetes	Hypocreales_fam_Incertae_sedis	Emericellopsis	NA	Col_0_dde2	*dde2*	Sordariomycetes;Emericellopsis;OTU_4
OTU_4	10.9746589	Ascomycota	Sordariomycetes	Hypocreales_fam_Incertae_sedis	Emericellopsis	NA	Ler_0_aba3_2	*aba3_2*	Sordariomycetes;Emericellopsis;OTU_4
OTU_418	8.656062204	Basidiomycota	Agaricomycetes	Ceratobasidiaceae	NA	unidentified	Col_0_etr1_3	*etr1_3*	Agaricomycetes;NA;OTU_418
OTU_42	-11.03442474	Ascomycota	Sordariomycetes	Hypocreales_fam_Incertae_sedis	Emericellopsis	NA	Col_0_etr1_3	*etr1_3*	Sordariomycetes;Emericellopsis;OTU_42
OTU_47	-6.589989262	Ascomycota	Sordariomycetes	Bionectriaceae	Clonostachys	NA	Col_0_fad3_2	*fad3_2*	Sordariomycetes;Clonostachys;OTU_47
OTU_53	-3.397046911	Ascomycota	Pezizomycetes	Pyronemataceae	NA	unidentified	Col_0_etr1_3	*etr1_3*	Pezizomycetes;NA;OTU_53
OTU_53	-8.6133535	Ascomycota	Pezizomycetes	Pyronemataceae	NA	unidentified	Col_0_35S_ERF	*35S_ERF*	Pezizomycetes;NA;OTU_53

OTU_53	-3.533603901	Ascomycota	Pezizomycetes	Pyronemataceae	NA	unidentified	Col_0_fad7_1fad8_1	*fad7_1fad8_1*	Pezizomycetes;NA;OTU_53
OTU_56	-6.83349302	Basidiomycota	Tremellomycetes	Tetragoniomycetaceae	Tetragoniomyces	Tetragoniomyces_uliginosus	Col_0_fad3_2	*fad3_2*	Tremellomycetes;Tetragoniomyces;OTU_56
OTU_67	-7.206670744	Basidiomycota	Tremellomycetes	Piskurozymaceae	Solicoccozyma	NA	Col_0_fad3_2	*fad3_2*	Tremellomycetes;Solicoccozyma;OTU_67
OTU_74	5.096039468	Olpidiomycota	Olpidiomycetes	Olpidiaceae	Olpidium	Olpidium_brassicae	Col_0_fad3_2	*fad3_2*	Olpidiomycetes;Olpidium;OTU_74
OTU_75	-7.963377376	Basidiomycota	Tremellomycetes	unidentified	NA	unidentified	Col_0_dde2	*dde2*	Tremellomycetes;NA;OTU_75
OTU_75	-4.377698948	Basidiomycota	Tremellomycetes	unidentified	NA	unidentified	Col_0_fad7_1fad8_1	*fad7_1fad8_1*	Tremellomycetes;NA;OTU_75
OTU_79	-6.568431136	Ascomycota	Ascomycota (Unknown Class)	NA	NA	NA	Col_0_fad3_2	*fad3_2*	Ascomycota (Unknown Class);NA;OTU_79
OTU_85	6.678709828	Ascomycota	Sordariomycetes	Nectriaceae	Dactylonectria	NA	Ler_0_aba3_2	*aba3_2*	Sordariomycetes;Dactylonectria;OTU_85
